# Genetic Diversity and Population Structure of Mesoamerican Jaguars (*Panthera onca*): Implications for Conservation and Management

**DOI:** 10.1371/journal.pone.0162377

**Published:** 2016-10-26

**Authors:** Claudia Wultsch, Anthony Caragiulo, Isabela Dias-Freedman, Howard Quigley, Salisa Rabinowitz, George Amato

**Affiliations:** 1 Sackler Institute for Comparative Genomics, American Museum of Natural History, New York, NY 10024, United States of America; 2 Public Health Research Institute, Rutgers University, Newark, New Jersey, United States of America; 3 Panthera, New York, NY 10018, United States of America; Embrapa, BRAZIL

## Abstract

Mesoamerican jaguars (*Panthera onca*) have been extirpated from over 77% of their historic range, inhabiting fragmented landscapes at potentially reduced population sizes. Maintaining and restoring genetic diversity and connectivity across human-altered landscapes has become a major conservation priority; nonetheless large-scale genetic monitoring of natural populations is rare. This is the first regional conservation genetic study of jaguars to primarily use fecal samples collected in the wild across five Mesoamerican countries: Belize, Costa Rica, Guatemala, Honduras, and Mexico. We genotyped 445 jaguar fecal samples and examined patterns of genetic diversity and connectivity among 115 individual jaguars using data from 12 microsatellite loci. Overall, moderate levels of genetic variation were detected (*N*_*A*_ = 4.50 ± 1.05, *A*_*R*_ = 3.43 ± 0.22, *H*_*E*_ = 0.59 ± 0.04), with Mexico having the lowest genetic diversity, followed by Honduras, Guatemala, Belize, and Costa Rica. Population-based gene flow measures (*F*_*ST*_ = 0.09 to 0.15, D_est_ = 0.09 to 0.21), principal component analysis, and Bayesian clustering applied in a hierarchical framework revealed significant genetic structure in Mesoamerican jaguars, roughly grouping individuals into four genetic clusters with varying levels of admixture. Gene flow was highest among Selva Maya jaguars (northern Guatemala and central Belize), whereas genetic differentiation among all other sampling sites was moderate. Genetic subdivision was most pronounced between Selva Maya and Honduran jaguars, suggesting limited jaguar movement between these close geographic regions and ultimately refuting the hypothesis of contemporary panmixia. To maintain a critical linkage for jaguars dispersing through the Mesoamerican landscape and ensure long-term viability of this near threatened species, we recommend continued management and maintenance of jaguar corridors. The baseline genetic data provided by this study underscores the importance of understanding levels of genetic diversity and connectivity to making informed management and conservation decisions with the goal to maintain functional connectivity across the region.

## Introduction

Over the last 100 years, jaguars (*Panthera onca*) have been extirpated from over 54% of their historic range [1–3]. These large-bodied and wide-ranging felids occur at low densities, depend on forest habitat, and are thereby negatively affected by changes in habitat connectivity and heterogeneity (e.g., [4, 5]). Habitat loss and fragmentation have the potential to limit movement in jaguars and consequently genetic connectivity, thus constituting two of the most significant threats to jaguars’ long-term survival (e.g., [1, 6–8]). Fragmented populations are more likely to experience genetic drift caused by small effective population sizes and reduced gene flow, leading to a decrease in genetic diversity and an increase in genetic structure (i.e., genetic differentiation, spatial distribution of genetic diversity), which negatively impacts short- and long-term persistence of wild populations (e.g., [9]). Low levels of genetic diversity decrease reproductive fitness, resistance to disease, and generally lower adaptive potential [10, 11]. In addition, jaguars have become increasingly threatened by illegal hunting, human-wildlife conflict, disease, overhunting of their prey base, and generally suffer from a lack of knowledge concerning their status in the wild in certain regions (e.g., [5, 12]). Consequently, jaguars are classified as ‘near-threatened’ under the International Union for Conservation of Nature (IUCN), declared as an ‘endangered’ species under the U.S. Endangered Species Act (ESA), and listed under Appendix I by the Convention on International Trade in Endangered Species of Wild Fauna and Flora (CITES) [12].

The situation is especially concerning in Mesoamerica, where jaguars occupy only 33% of their former range, and 75% of existing populations inhabit fragmented landscapes at potentially reduced population sizes [2, 13]. Mesoamerica has experienced one of the highest deforestation rates worldwide caused by illegal logging, drug trafficking, and agricultural development (e.g., [14, 15]). Additionally, shipping corridors such as the Panama Canal and the current construction of a similar waterway in Nicaragua, pose further threats to connectivity of Central American wildlife such as forest-dependent jaguars (e.g., [16]. Large-scale conservation efforts, including the Mesoamerican Biological Corridor (MBC, [17]) and Panthera’s Jaguar Corridor Initiative (JCI, [6]) have been implemented in response to these threats, seeking to maintain connectivity between protected areas for wide-ranging wildlife species. The JCI aims to identify, prioritize, assess, and consequently protect and restore Jaguar Conservation Units (JCUs) and movement corridors between these core jaguar populations to preserve connectivity across the Mesoamerican landscape and beyond [6, 18].

Large-scale conservation strategies, which are instrumental to protect broadly distributed species such as jaguars (e.g., [2, 18, 19]), need to incorporate genetic monitoring of wild populations to fully understand how these species respond to environmental changes and increasing levels of anthropogenic impacts. Evaluating genetic connectivity by identifying genetic clusters (e.g., populations), and quantifying levels of genetic diversity within populations and gene flow between them, is crucial when prioritizing conservation and management efforts (e.g., [9, 20]). Nonetheless, the implementation of large-scale genetic surveys is challenging and the difficulty in obtaining DNA samples from wild populations has limited the number of genetic studies in jaguars [21–24]. In recent years, the application of noninvasive genetic sampling (e.g., fecal DNA), scat detector dogs, and optimization of field and laboratory methods have helped increase sample sizes for difficult-to-study and elusive target species such as many wild felids (e.g., [23, 25, 26]). However, large-scale genetic studies often still suffer from low sample sizes, making it difficult to detect cryptic genetic structure at differing spatial scales. Examining levels of population subdivision on fine- and large spatial scales increases our understanding of historical, ecological, and behavioral processes driving genetic connectivity, which represents crucial information to formulate effective conservation and management efforts.

Previous large-scale genetic studies suggested that jaguars exhibit relatively high levels of genetic diversity and weak population structure across their range, rejecting subdivision of jaguars into subspecies [21, 22], which contradicts earlier studies identifying eight to sixteen subspecies based on skull morphology (e.g., [3, 27, 28]). Eizirik, Kim [21] did not detect strong genetic differentiation for jaguars across their range, but suggested subdivision into four incompletely isolated phylogeographic groups (Mexico and Guatemala, southern Central America, northern South America, and southern South America). Ruiz-García, Vásquez [22] could not support these findings, but concluded that genetic diversity levels were lower for Guatemalan jaguars in comparison to most other South American populations, thus making Mesoamerican jaguars a higher conservation priority and in need of further genetic surveys. Sample sizes for Mesoamerican jaguars in these range-wide studies were also low (13 individuals, Eizirik, Kim [21]; 10 individuals, Ruiz-García, Vásquez [22]). In recent years, however, fine-scale genetic monitoring efforts focusing on jaguars [23, 24, 29] showed evidence for genetic differentiation, suggesting that human-dominated landscapes surrounding remaining jaguar habitat potentially limited gene flow of jaguars at a local scale.

This current study represents the first regional genetic survey investigating genetic diversity, gene flow, and dispersal patterns for Mesoamerican jaguars using DNA from scat samples collected in the wild. We characterized levels of neutral genetic diversity and the degree of genetic connectivity for jaguars at several sampling sites across five Mesoamerican countries (Belize, Costa Rica, Guatemala, Honduras, Mexico). We also evaluated the assumption that Mesoamerican jaguars are one large panmictic population, exhibiting low levels of genetic differentiation, as suggested by earlier studies. The long-term goal of this study is to support regional conservation and management efforts such as JCI and MBC by providing genetic baseline data, assessing functional connectivity of movement corridors (“genetic ground-truthing”), and making recommendations for the maintenance of genetic connectivity and long-term persistence of Mesoamerican jaguars on a regional scale.

## Materials and Methods

### Ethics statement

A review from the ethics committee was not required, as the fieldwork only involved noninvasive genetic sampling of fecal samples. Permits and necessary permissions for collection of felid scat samples were obtained from the Belize Forest Department, the Ministry of Environment and Energy of Costa Rica, the National Council of Protected Areas of Guatemala, the National Institute for Conservation and Forest Development, Protected Areas, and Wildlife of Honduras, and the Secretariat of Environment and Natural Resources of Mexico.

### Study sites and sampling information

**Fig 1:**
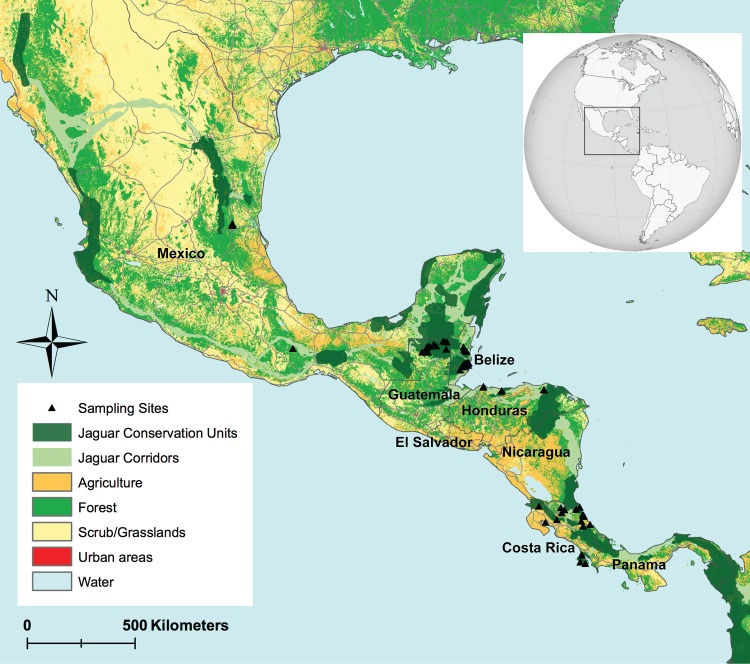
Sampling sites of jaguars across five Mesoamerican countries. Black triangles represent individual jaguar samples collected across five Mesoamerican countries (Mexico, Guatemala, Belize, Honduras, Costa Rica). Spatial data representing jaguar conservation units and movement corridors were obtained from Panthera.

Fecal samples were collected opportunistically in the field at several sampling sites across five Mesoamerican countries, including Belize (Central Belize Corridor Area, 17°30’ N, 88°34’ W; Cockscomb Basin Wildlife Sanctuary, 16°48’ N, 88°37’ W), Costa Rica (Tortuguero National Park, 10°26’ N, 83°30’ W; northern Talamanca, 10°00’ N, 83°30’ W; Osa Peninsula, 8°43’ N, 83°22’ W), Guatemala (Laguna del Tigre National Park, 17°34’ N, 90°40’ W; Maya Biosphere Reserve, 17°28’ N, 89°51’ W; Mirador R**í**o-Azul National Park, 17°48’ N, 89°15’ W), Honduras (Río Plátano Biosphere Reserve, 15°31’ N, 84°46’ W; Pico Bonito National Park, 15°37’ N, 86°51’ W; Jeanette Kawas National Park, 15°50’ N, 87°40’ W), and Mexico (Sierra del Abra Tanchipa Biosphere Reserve, 22°15’ N, 98°56’ W; Sierra Mixe, Oaxaca, 17°25’ N, 96°22’ W) (Fig 1). Field sampling efforts in Costa Rica were supported by the use of a professionally trained scat detector dog. The sampling sites were primarily located in low-lying regions with subtropical and tropical climates and a high diversity of habitat types. All sampling sites are part of or in close proximity to the Mesoamerican Biological Corridor and Panthera’s JCUs. Fecal samples were preserved using silica beads and stored at room temperature prior to DNA extraction. Geographic locations of fecal samples were recorded using a GPS unit.

### DNA extraction, PCR and genotyping

Genomic DNA was extracted from fecal samples using the QIAamp DNA Stool Mini Kit (Qiagen, Inc., Valencia, CA) with small protocol modifications and species were identified using four mitochondrial gene regions [30]. For individual identification, we used 12 polymorphic nDNA microsatellite loci [31, 32] arranged in five polymerase chain reaction (PCR) multiplex reactions (multiplex 1 –*FCA032*, *FCA100*, *FCA124*; multiplex 2 –*FCA126*, *FCA212*, *FCA229*; multiplex 3 –*FCA096*, *FCA132*, *FCA275*; multiplex 4 –*FCA075*, *FCA208*; multiplex 5 –*FCA225*). At first, multiplex reactions of 20 μL consisted of reagents as described in Caragiulo, Kang [33] and utilized 3.0–5.0 μL of genomic DNA. Starting in 2014, reagents and DNA in multiplex reactions were optimized and reduced to 10–10.25 μL volumes. For additional details on updated PCR reactions and thermocycling conditions for multiplex 1–5, see S1 Materials and Methods.

To identify gender, we used ZF-1F/ZFX-1R and ZFY-2F/ZF-2R [34] and Zn/Amel DNA markers [35]. PCR reactions for the ZF-1F/ZFX-1R and ZFY-2F/ZF-2R primer set were conducted and analyzed as described in Caragiulo, Kang [33]. Thermocycling conditions for the ZF-1F/ZFX-1R and ZFY-2F/ZF-2R primers followed [34] except for 13 cycles during the touchdown sequence, and annealing at 53°C for 45 s. For the Zn/Amel primers [35], PCR reactions and thermocycling conditions were conducted following protocols described by Wultsch, Waits [23]. A PCR negative was included in each PCR group to control for contamination. Primers were fluorescently labeled and PCR products were visualized using an ABI 3730xl DNA analyzer (Applied Biosystems™, Carlsbad, CA). Scoring of alleles was conducted using GENEMAPPER, version 5.0 (Applied Biosystems™, Carlsbad, CA). To finalize consensus genotypes and minimize genotyping error, we employed a multi-tube approach [36] with a minimum of 4 repetitions for each microsatellite multiplex and jaguar sample. To confirm individual identification and assess the resolving power of the 12 microsatellite loci, we calculated probability of identity values (P_(ID)_, the probability of identity; P_(ID)sibs_, the probability of identity between siblings), as suggested by Mills, Citta [37] and Waits, Luikart [38] using GenAlEX, version 6.5 [39]. In addition, we quantified nDNA amplification success (number of successful PCRs divided by total number of PCRs across all loci) and genotyping error (allelic dropout, ADO; false allele, FA) rates for microsatellites using *ConGenR* [40] in R, version 3.1.3 [41]. FA rates were calculated for all consensus genotypes and ADO rates were estimated only for heterozygous genotypes.

### Genetic diversity, Hardy-Weinberg equilibrium, and linkage disequilibrium

Standard indices of genetic diversity, including number of alleles (*N*_*A*_), observed (*H*_*O*_) and expected heterozygosities (*H*_*E*_) for each and across all microsatellite loci and sampling sites, were estimated using *diveRsity*, version 1.9.89 [42] in R, version 3.1.3 [41]. We also calculated rarified allelic richness (*A*_*R*_) and inbreeding coefficients (*F*_*IS*_) with *hierfstat* [43] in R, version 3.1.3 [41]. 95% confidence intervals of multi-locus inbreeding coefficients were estimated (1,000 bootstraps) over 12 loci. Conformation to Hardy-Weinberg equilibrium (HWE) and linkage disequilibrium (LD) was tested performing exact tests in GENEPOP, version 4.1 [44] with default settings for Markov chain parameters (dememorization = 1000; batches = 100; Markov chain Monte Carlo [MCMC] iterations per batch = 1000). We screened each microsatellite locus across sampling sites for the occurrence of null alleles using MICROCHECKER, version 2.2.3 [45]. Statistical differences between groups were evaluated using Kruskal-Wallis rank-sum tests in R, version 3.1.3 [41]. Sequential Bonferroni correction [46] was applied to correct significance levels for multiple tests performed simultaneously.

### Genetic structure

We quantified genetic structure using individual- and population-based analysis approaches at both fine and regional spatial scales. First, to assess contemporary gene flow, non-spatial Bayesian clustering using STRUCTURE, version 2.3.4 [47, 48] was applied to infer population structure by probabilistically assigning individuals to a number of genetic groups/clusters (*K*). A series of 10 independent runs per *K* (ranging from 1 to 10) was conducted using the admixture model with correlated allele frequencies, sampling locations as prior (LOCPRIOR), and 2,000,000 MCMC iterations after a burn-in of 200,000 replicates. We chose the most likely *K* value by calculating the mean posterior probability, mean L(*K*) [47] and delta *K* (Δ*K*) statistic [49] for each *K* using POPHELPER [50] in R, version 3.2.4 [41]. To examine hierarchical genetic structure within the genetic clusters identified through STRUCTURE, we repeated Bayesian clustering analysis until no additional genetic subdivision was detected (e.g., [51, 52]). Since the Bayesian clustering algorithm in STRUCTURE assumes unrelatedness among sampled individuals [47], we also identified closely related jaguars (parent-offspring, full siblings) with ML-RELATE [53], and repeated the STRUCTURE analysis without including closely related individuals. At last, individual genotypes were assigned to distinct genetic clusters by summarizing the percentage of genotypes' ancestry (*Q*; scores ≥70% indicate assignment to a genetic cluster; scores <70% and >30% indicate admixed ancestry). To complement the STRUCTURE analysis, we also conducted a principal component analysis (PCA) and identified clusters of genetically similar individuals without making assumptions regarding HWE and LD, as implemented in *adegenet*, version 1.4.2 [54] using R, version 3.1.3 [41].

Furthermore, we examined contemporary gene flow (i.e. within the past few generations) by identifying first-generation dispersers (*F*_*0*_) through assignment tests implemented in GENECLASS, version 2.0 [55]. The probability of individual genotypes coming from each sampling location was calculated by running simulations with 10,000 individuals and a Type I error level of 0.01. The likelihood estimation was based on *Lhome*/*Lmax* (ratio of *L_home* to the highest likelihood value among all available population samples including the population where the individual was sampled, *L_max*), applying the algorithm of Rannala and Mountain [56] in combination with the MCMC resampling method of Paetkau, Slade [57].

The presence of genetic structure was also assessed by calculating population-based gene flow estimates such as pairwise *F*_*ST*_ [58] and D_est_ [59] values with 95% confidence intervals (10,000 bootstraps) between genetic clusters identified by STRUCTURE (cluster 1—Mexico, cluster 2 –Guatemala and Belize, cluster 3 –Honduras, cluster 4—Costa Rica) using *diveRsity*, version 1.9.89 [42] in R, version 3.1.3 [41]. In addition, to examine the effect of spatial scale on the genetic structure analyses, we conducted an analysis of molecular variance (AMOVA, [60]), estimating the degree of genetic differentiation between-group and within-group at several hierarchical levels through calculating variance components and Phi values (Φ, statistics analogous to Wright’s *F*-statistics) with *ade4*, version 1.7–4 [61] in R, version 3.1.3 [41]. Different *a priori* hypotheses about hierarchical genetic divergence were tested, including subdivision into historically defined subspecies (scenario A: *Panthera o*. *hernandesii*, *Panthera o*. *veraecrucis*, *Panthera o*. *goldmani*, *Panthera o*. *centralis*; due to low sample sizes *Panthera o*. *hernandesii* and *Panthera o*. *veraecrucis* were defined as one group), JCUs covered in this study (scenario B: JCU 2 –Sierra Madre Oriental, Mexico; JCU 5 –Istmo de Tehuantepec, Mexico; JCU 8 –Selva Maya (Mexico, Guatemala, Belize); JCU 14 –west-central Belize; JCU 19 –Corazón Biosphere, Honduras; JCU 20 –Río Indio Maíz, Costa Rica; JCU 21 –northwestern Costa Rica; JCU 25 –Osa Peninsula, Costa Rica), and genetic clusters identified through Bayesian clustering in STRUCTURE (scenario C: cluster 1—Mexico, cluster 2 –Guatemala and Belize, cluster 3 –Honduras, cluster 4—Costa Rica). To determine significance values for Φ statistics, randomization tests were applied using 1,000 permutations.

Finally, isolation-by-distance (IBD) patterns were examined to determine whether a significant correlation existed between pairwise codominant genotypic and geographic (Euclidean) distances by performing simple Mantel tests (1,000 permutations) across fine- and large spatial scales with *ecodist*, version 1.2.9 [62] in R, version 3.1.3 [41]. In addition, we conducted a spatial autocorrelation analysis in GenAlEx, version 6.5 [39], to assess genetic similarity between pairs of individuals at several distance classes, and by doing so examine the spatial extent of positive genetic structure. A significant positive autocorrelation means that individuals at a given distance class are genetically more similar than expected by chance. We determined the significance of spatial autocorrelation coefficients (*r*), at each distance class via permutation (10,000 simulations) and bootstrapping (1,000 repeats). Spatial autocorrelation patterns were graphically visualized using correlograms.

## Results

### Microsatellite genotyping

We genotyped a total of 445 jaguar scats collected across five Mesoamerican countries (Belize [*n* = 297], Costa Rica [*n* = 50], Guatemala [*n* = 24], Honduras [*n* = 34], Mexico [*n* = 40]). We identified 115 individual jaguars (74 males, 11 females, 30 sex unknown; Table 1). Cumulative P_(ID)_ and P_(ID)sibs_ estimates were 3.36E-11 and 5.82E-05, which confirms high resolving power for identifying individual jaguars using this set of microsatellites. Overall nDNA PCR amplification success rate for microsatellites was 60.4%. Cumulative mean genotyping error rates were 4.9% for false alleles and 17.9% for allelic dropout.

**Table 1:** DNA sample summary. Number of individual jaguars (*n*) and number of males and females detected across five Mesoamerican countries (Belize, Costa Rica, Guatemala, Honduras, Mexico).

Country	*n*	*Males*	*Females*	*Sex Unknown*
Mexico	7	6	1	0
Guatemala	15	12	0	3
Belize	50	41	3	6
Honduras	7	6	0	1
Costa Rica	36	9	7	20
**Total**	115	74	11	30

### Genetic diversity, Hardy-Weinberg equilibrium and linkage disequilibrium

Overall, Mesoamerican jaguars exhibited moderate to high levels of genetic diversity across all loci, with *N*_*A*_ of 4.50 (±1.05, SD), rarified *A*_*R*_ of 3.43 (±0.22, SD), *H*_*O*_ of 0.59 (±0.06, SD), and *H*_*E*_ of 0.59 (±0.04, SD) (Table 2 and S1 Table). Genetic diversity estimates were highest for jaguars in Costa Rica, followed by Belize, Guatemala, Honduras, and Mexico (Table 2 and S1 Table), but did not differ significantly across sampling sites (Kruskal–Wallis rank-sum tests: *A*_*R*_, *H* = 2.35, *P* = 0.673; *H*_*e*_, *H* = 4.47, *P* = 0.346;) with the exception of *N*_*A*_ *(H* = 13.56, *P* = 0.010). The global inbreeding coefficient was 0.05 (±0.09, SD), ranging from -0.08 to 0.13 at different sampling localities (Table 2 and S1 Table). We detected null alleles in *FCA075* and *FCA212* in Belizean jaguars (S2 Table). However, since null allele frequencies were low and no signs of null alleles were detected for these two loci at any of the other sampling localities, we kept *FCA075* and *FCA212* in the analysis.

**Table 2:** Summary statistics of genetic diversity for Mesoamerican jaguars.

Country	*n*	*N*_*A*_	*A*_*R*_	*H*_*O*_	*H*_*E*_	*F*_*IS*_ (95% CI [LL, UL])
Mexico	7	3.25	3.14	0.52	0.54	0.13 (0.00, 0.29)
Guatemala	15	4.33	3.35	0.67	0.60	-0.08 (-0.15, 0.00)
Belize	50	5.00	3.38	0.54	0.60	0.12 (0.03, 0.18)
Honduras	7	3.92	3.55	0.62	0.58	-0.01 (-0.13, 0.18)
Costa Rica	36	6.00	3.72	0.62	0.64	0.05 (0.01, 0.11)
Total Mean		4.50	3.43	0.59	0.59	0.05
*SD*		1.05	0.22	0.06	0.04	0.09

Genetic variability was assessed based on 115 jaguar individuals genotyped at 12 microsatellite loci collected across five Mesoamerican countries (Belize, Costa Rica, Guatemala, Honduras, Mexico). Summary includes number of individuals (*n*), number of alleles (*N*_*A*_), rarefied allelic richness (*A*_*R*_), observed heterozygosity (*H*_*O*_), expected heterozygosity (*H*_*E*_), inbreeding coefficient (*F*_*IS*_) and 95% confidence intervals (CI; 1,000 bootstraps) with lower (LL) and upper (UL) limits. *SD*, standard deviation.

Departure from Hardy-Weinberg equilibrium was significant in two cases (*FCA075* in Belize, *P* = 0.001; *FCA126* in Costa Rica, *P* = 0.003) after applying sequential Bonferroni correction (S2 Table). LD was not observed at any locus pairs after applying sequential Bonferroni corrections, suggesting that all loci are genetically independent.

### Genetic structure

**Fig 2:**
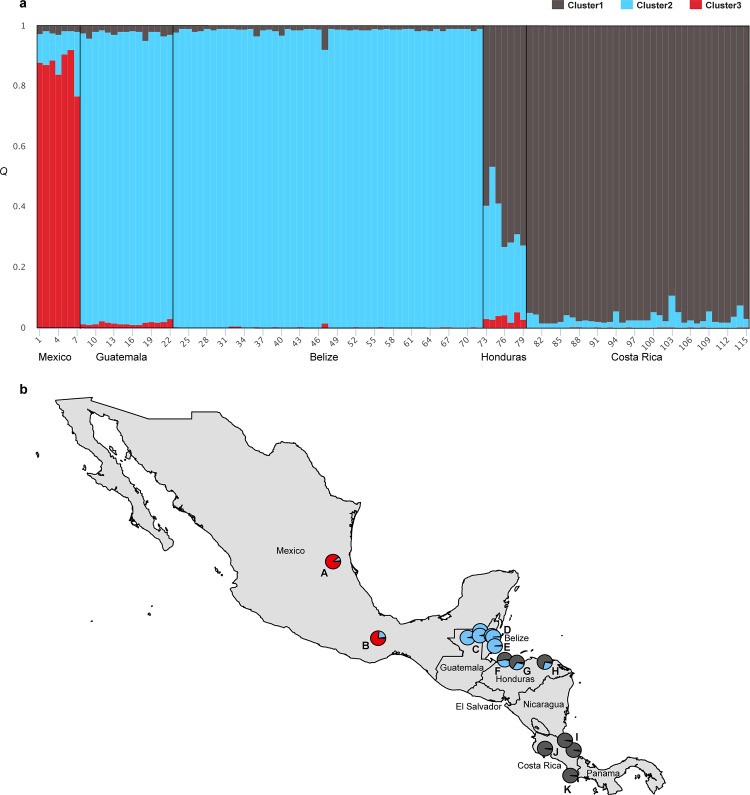
Genetic structure in Mesoamerican jaguars. Results were obtained from Bayesian clustering analysis (*K* = 3) using STRUCTURE, version 2.3.4 [47] and genotype data from 12 microsatellite loci for jaguars (*n* = 115) sampled across five Mesoamerican countries (Belize, Costa Rica, Guatemala, Honduras, Mexico). (a) STRUCTURE barplot—vertical bars represent individuals and the color of each bar visualizes the % of membership (*Q*) the individual belongs to the genetic clusters (*K*) identified. (b) Pie charts represent fractions of *Q* for groups of jaguar individuals studied across different sampling sites (A, Sierra del Abra Tanchipa Biosphere Reserve, Mexico; B, Sierra Mixe, Oaxaca, Mexico; C, northern Guatemala [Laguna del Tigre National Park, Maya Biosphere Reserve, Mirador R**í**o-Azul National Park]; D, Central Belize Corridor Area, Belize; E, Cockscomb Basin Wildlife Sanctuary, Belize; F, Jeanette Kawas National Park, Honduras; G, Pico Bonito National Park, Honduras; H, Río Plátano Biosphere Reserve, Honduras; I, northeastern Costa Rica [Tortuguero National Park, Barra del Colorado Wildlife Refuge, and Barbilla National Park]; J, several sites across northern and northwestern Costa Rica; K, Corcovado National Park, Costa Rica.

**Fig 3:**
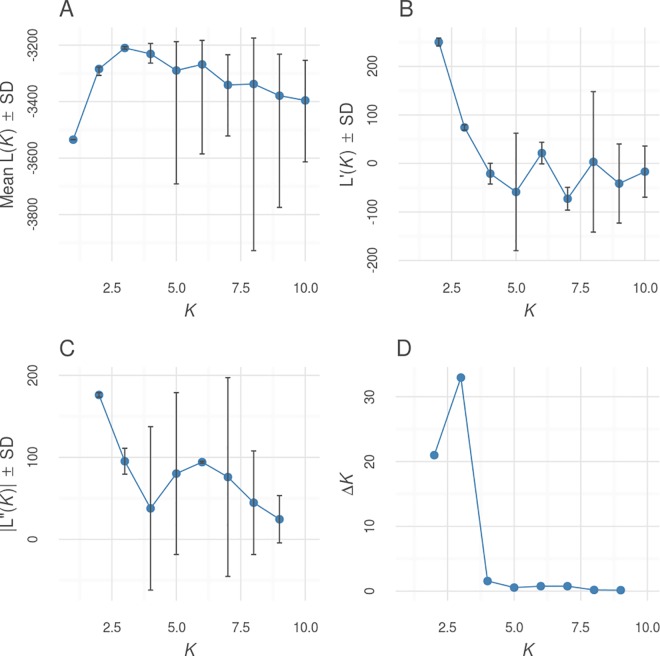
Results of the global STRUCTURE analysis in Mesoamerican jaguars. The optimal number of genetic clusters (*K*) in Mesoamerican jaguars (*n* = 115) applying Bayesian clustering methods in STRUCTURE, version 2.3.4 [47] was chosen based on posterior probabilities (mean L(*K*); A) and delta *K* (Δ*K*, mean (|L”(*K*)|)/SD(L(*K*)); D) for each *K* value. SD, standard deviation; L’(*K*), mean rate of change of the likelihood distribution (B); |L”(*K*)|, absolute value of the 2^nd^ order rate of change of the likelihood distribution (C).

**Fig 4:**
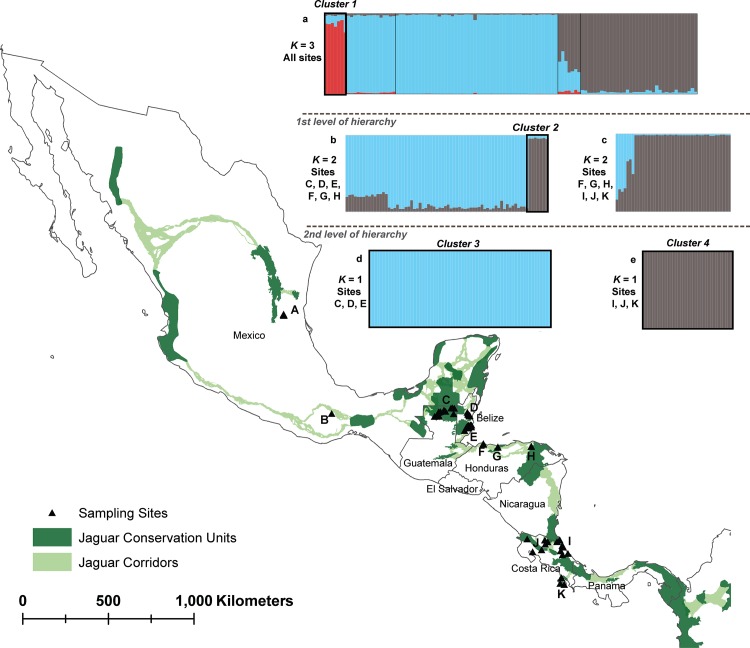
Hierarchical genetic structure in Mesoamerican jaguars. Bayesian clustering analysis was conducted for all jaguar samples (a, *n* = 115) at a first (b, *n* = 72; c, *n* = 42) and a second hierarchy level (d, *n* = 65; e, *n* = 36) using STRUCTURE, version 2.3.4 [47]. Hierarchical structure analysis identified four genetic clusters (cluster 1: sites A and B; cluster 2: sites F, G, and H; cluster 3: C, D, and E; cluster 4: I, J, and K). A, Sierra del Abra Tanchipa Biosphere Reserve, Mexico; B, Sierra Mixe, Oaxaca, Mexico; C, northern Guatemala (Laguna del Tigre National Park, Maya Biosphere Reserve, Mirador R**í**o-Azul National Park); D, Central Belize Corridor Area, Belize; E, Cockscomb Basin Wildlife Sanctuary, Belize; F, Jeanette Kawas National Park, Honduras; G, Pico Bonito National Park, Honduras; H, Río Plátano Biosphere Reserve, Honduras; I, northeastern Costa Rica (Tortuguero National Park, Barra del Colorado Wildlife Refuge, and Barbilla National Park); J, several sites across northern and northwestern Costa Rica; K, Corcovado National Park, Costa Rica. *K*, number of genetics clusters inferred in STRUCTURE.

**Fig 5:**
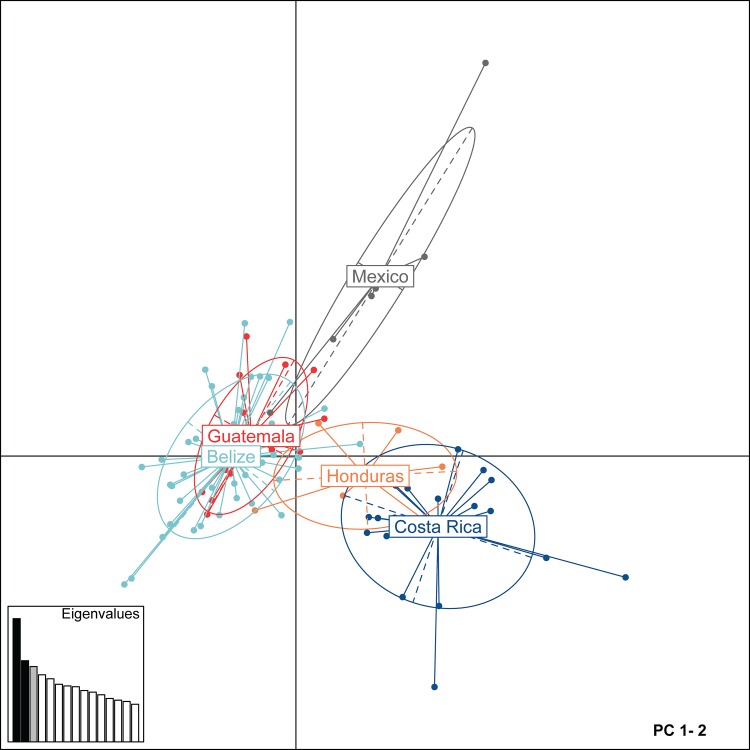
Principal component analysis (PCA) based on Mesoamerican jaguar genotypes. Scatterplot visualizes the first two principal components. Points represent individual genotypes, and geographical groups of jaguars sampled across five Mesoamerican countries (Belize, Costa Rica, Guatemala, Honduras, Mexico) are 95% inertia ellipses in different colors. Inserted barchart shows eigenvalues with corresponding components in dark grey. PC, principal component.

Non-spatial Bayesian clustering analysis in STRUCTURE for all Mesoamerican jaguars suggested subdivision into three genetic clusters (*K* = 3) with varying levels of genetic admixture (Figs 2 and 3). For *K* = 3, the identified genetic clusters roughly corresponded to main geographic regions (north–cluster 1—Mexico; central–cluster 2—Guatemala, Belize, and Honduras; south–cluster 3—Honduras and Costa Rica) sampled across Mesoamerica and varied in levels of genetic admixture (Fig 2). In Honduras, we detected 3 jaguars with admixed ancestry partially assigned to cluster 2 and 3, and 4 jaguars fully assigned to cluster 3 (*Q* > 0.7). Further hierarchical clustering analysis of jaguars sampled across the Selva Maya region (northern Guatemala and central Belize) and Honduras showed evidence for genetic differentiation (*K* = 2): Selva Maya jaguars clustered separately from Honduran jaguars with overall high assignment membership values for all individuals (*Q* > 0.7) (Fig 4B and S1A Fig). For Honduran and Costa Rican jaguars, we also detected *K* = 2, suggesting the presence of two genetic clusters with evidence for genetic admixture (Fig 4C and S1B Fig). Costa Rican jaguars were all assigned to the first cluster and Honduran jaguars exhibited admixed ancestry between both clusters detected. For Selva Maya jaguars, the highest Δ*K* was observed at *K* = 8, but due to genetic homogeneity across all samples (symmetric assignment of individuals to all genetic clusters), we concluded that low genetic structuring (*K* = 1) was evident (Fig 4D and S1C Fig). Furthermore, Bayesian clustering did not detect genetic substructure (*K* = 1) when Costa Rican jaguars were assessed separately (Fig 4E and S1D Fig). Additional Bayesian clustering analysis in STRUCTURE excluding closely related jaguar individuals from the analysis corroborated our previous findings and confirmed that the number of genetic clusters was not overestimated due to the presence of closely related jaguars (S2 Fig). In accordance with the Bayesian clustering analysis in STRUCTURE, the PCA plot showed that Selva Maya jaguars were clearly clustered into one group, while jaguars sampled in Mexico, Honduras, and Costa Rica formed separate genetic clusters, suggesting moderate genetic differentiation (Fig 5).

Estimation of contemporary gene flow through likelihood-based assignment tests conducted in GENECLASS detected two putative *F*_*0*_ dispersers among sampling sites across Mesoamerica. One *F*_*0*_ disperser was detected in southern Mexico at Sierra Mixe in Oaxaca, indicating gene flow from the Guatemala sampling sites. The second *F*_*0*_ disperser sampled at the Río Plátano Biosphere Reserve in southeastern Honduras was genetically assigned to sampling sites in Costa Rica (Table 3). Both *F*_*0*_ dispersers were male.

**Table 3:** First-generation migrant analysis indicating dispersers in Mesoamerican jaguars.

			GENECLASS *F*_*0*_ migrant			-log(L) by sampling site				
Sample ID	Sex	Origin	log(L_home/L_max)	A	*P*	MX	GTA	BZ	HON	CR
Jaguar01	M	MX	1.70	GTA	0.001	9.95	8.25	8.28	14.28	13.30
Jaguar02	M	HON	3.78	CR	0.005	17.66	18.79	18.62	18.61	14.83

First-generation migrants (*F*_*0*_) were identified for Mesoamerican jaguars (*n* = 115) across sampling sites in Mexico (MX), northern Guatemala (GTA), central Belize (BZ), Honduras (HON), and Costa Rica (CR) using likelihood computations (L_ home/L_max) in GENECLASS, version 2.0 [55]. Origin, geographical sampling location of individual felid; A, genetic assignment of individual felid to geographic origin cluster; *P*, *P*-value refers to the detection of *F*_*0*_ dispersers (*P* < 0.05 indicates a potential *F*_*0*_).

Population-based gene flow estimates using pairwise *F*_*ST*_ and D_est_ values between sampling sites indicated moderate genetic differentiation, with *F*_*ST*_ values ranging from 0.09 to 0.15, and D_est_ values ranging from 0.09 to 0.21 (Table 4). The highest *F*_*ST*_ and D_est_ estimates were observed between jaguars detected in Mexico and all remaining sites, and between Selva Maya and Costa Rican jaguars. The AMOVA results testing three hypotheses for hierarchical genetic subdivision (scenario A: historically defined subspecies, scenario B: JCUs, and scenario C: genetic clusters identified with STRUCTURE) indicated that most of the variance (V) was attributable to genetic variation among the lowest hierarchical level, individuals within sampling sites (A: V = 71.7%, Φ = 0.28, *P* = 0.001; B: V = 75.5%, Φ = 0.24, *P* = 0.001; C: V = 71.6%, Φ = 0.28, *P* = 0.001) (Table 5). These results suggested that genetic variation resided primarily within sampling localities rather than among them, which was consistent at all scales.

**Table 4:** Genetic differentiation in Mesoamerican jaguars.

*Panthera onca* (*n* = 115)	1	2	3	4
**1**	—	0.15 (0.06–0.26)	0.14 (0.05–0.27)	0.15 (0.06–0.26)
**2**	0.11 (0.02–0.24)	—	0.10 (0.05–0.16)	0.13 (0.08–0.19)
**3**	0.11 (0.03–0.24)	0.10 (0.03–0.21)	—	0.09 (0.04–0.13)
**4**	0.14 (0.05–0.27)	0.21 (0.13–0.29)	0.09 (0.01–0.21)	—

Pairwise *F*_*ST*_ [58] and *D*_*est*_ [59] estimates, with 95% confidence intervals (10,000 bootstraps) in brackets. Mesoamerican jaguar samples were grouped based on genetic clusters identified in STRUCTURE, version 2.3.4 [47] (1: cluster 1—Mexico, *n* = 7; 2: cluster 2—Guatemala and Belize, *n* = 65; 3: cluster 3—Honduras, *n* = 7; 4: cluster 4—Costa Rica, *n* = 36). *F*_*ST*_ estimates are above the diagonal, with pairwise *D*_*est*_ estimates below the diagonal. *n*, number of individuals.

**Table 5:** Hierarchical analysis of molecular variance (AMOVA) in Mesoamerican jaguars.

	Source of variation	d.f.	SS	VC	% Variance	Φ	*P*
**A**	Among historically defined subspecies	2	12.13	0.11	15.83	0.16	0.001
	Among sampling sites within hist. defined subspecies	96	65.13	0.09	12.49	0.15	0.001
	Among individuals within sampling sites	99	49.81	0.50	71.68	0.28	0.001
	Total	197	127.08	0.70	100.00		
**B**	Among JCUs	6	16.17	0.08	12.38	0.12	0.001
	Among sampling sites within JCUs	92	61.09	0.08	12.07	0.14	0.002
	Among individuals within sampling sites	99	49.81	0.50	75.54	0.24	0.001
	Total	197	127.08	0.67	100.00		
**C**	Among genetic clusters	3	14.22	0.12	16.96	0.17	0.001
	Among sampling sites within genetic clusters	95	63.05	0.08	11.42	0.14	0.002
	Among individuals within sampling sites	99	49.81	0.50	71.62	0.28	0.001
	Total	197	127.08	0.70	100.00		

The analysis was based on 115 jaguar individuals detected at several sampling sites in Mexico, Guatemala, Belize, Honduras, and Costa Rica and genotyped at 12 microsatellite loci. Different hypotheses for hierarchical genetic subdivision: historically defined subspecies for Mesoamerican jaguars (scenario A: *Panthera o*. *hernandesii*, *Panthera o*. *veraecrucis*, *Panthera o*. *goldmani*, *Panthera o*. *centralis*; due to low sample sizes *Panthera o*. *hernandesii* and *Panthera o*. *veraecrucis* were defined as one group), Jaguar Conservation Units (JCUs) included in this study [21] (scenario B: JCU 2 –Sierra Madre Oriental, Mexico; JCU 5 –Istmo de Tehuantepec, Mexico; JCU 8 –Selva Maya, Mexico, Guatemala, and Belize; JCU 14 –west-central Belize; JCU 19 –Corazón Biosphere, Honduras; JCU 20 –Río Indio Maíz, Costa Rica; JCU 21 –northwestern Costa Rica; JCU 25 –Osa Peninsula, Costa Rica), and genetic clusters identified with STRUCTURE, version 2.3.4 [47] (scenario C: cluster 1—Mexico, cluster 2 –Guatemala and Belize, cluster 3 –Honduras, cluster 4—Costa Rica) were tested. d.f., degrees of freedom; SS, sum of squares; VC, variance components; % Variance, proportion of variance attributed to the different levels in spatial hierarchy of Mesoamerican jaguars; Φ, Phi statistics analogous to Wright’s *F*-statistics; *P*, *P* values based on 1,000 permutations.

**Fig 6:**
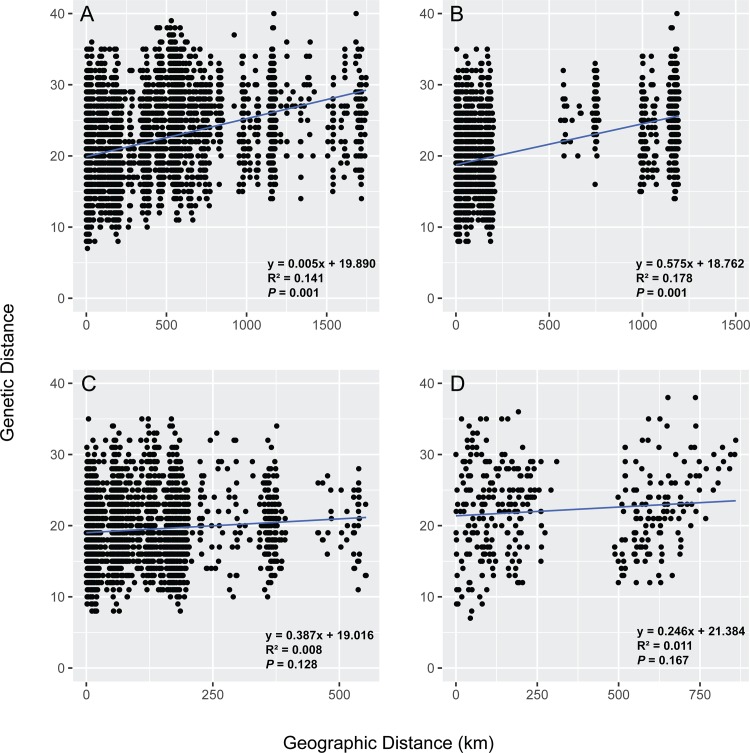
Regional and fine-scale isolation-by-distance patterns in Mesoamerican jaguars. Isolation-by-distance in Mesoamerican jaguars was assessed by plotting pairwise codominant genotypic distance calculated in GenAlEx, version 6.41 [39] versus pairwise Euclidean distances (km) across (A) all sampling sites, (B) Mexico, Guatemala, and Belize, (C) Guatemala, Belize, and Honduras, and (D) Honduras and Costa Rica. Statistical significance was assessed using simple Mantel tests in *ecodist*, version 1.2.9 [62] in R, version 3.1.3 [41]. Each point represents a pairwise comparison among individual jaguars.

**Fig 7:**
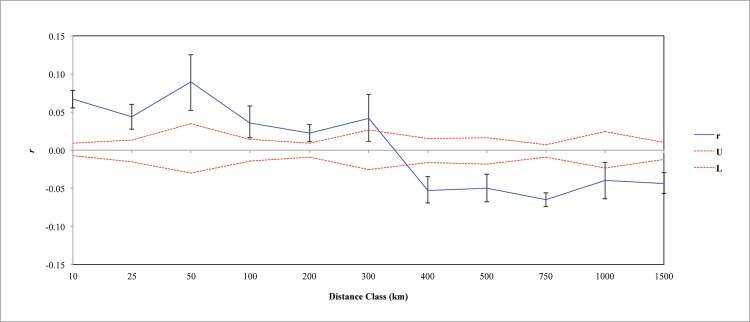
Spatial autocorrelation in Mesoamerican jaguars. Spatial correlogram for jaguars (*n* = 99) showing the genetic correlation coefficient (*r*) as a function of geographic distance across defined spatial distance classes. Dashed red lines represent upper (U) and lower (L) bounds of the null hypothesis of no spatial structure based on 10,000 random permutations. Error bars represent 95% confidence intervals about *r* based on 1,000 bootstraps.

Across all samples, we found a significant IBD pattern (*r* = 0.375, *P* = 0.001) (Fig 6A). Mantel tests performed on a finer geographic scale indicated a significant correlation between genetic and geographic distances for jaguars within Mexico, northern Guatemala, and central Belize (*r* = 0.422, *P* = 0.001) (Fig 6B). We did not observe significant correlations between genetic and geographic distances of jaguars collected across other areas (northern Guatemala, central Belize, and Honduras, *r* = 0.089, *P* = 0.128, Fig 6C; Honduras and Costa Rica, *r* = 0.104, *P* = 0.167, Fig 6D), verifying that geographic distance was not the main factor driving genetic differentiation at these spatial scales. We also detected IBD in the spatial autocorrelation analysis with significant positive correlation between individuals in the first five distance classes (10km, *r* = 0.057, *P* = 0.001; 25km, *r* = 0.034, *P* = 0.001; 50km, *r* = 0.079, *P* = 0.001; 100km, *r* = 0.026, *P* = 0.001; 200km, *r* = 0.012, *P* = 0.001), after which *r* decreased to become statistically indistinguishable from zero, as would be expected in the case of nonrandom genetic structure such that spatially proximal individuals are genetically more similar than spatially distant jaguars. The spatial autocorrelation analysis also revealed an x-intercept of *r* at ~ 340 km, indicating the extent of positive genetic structure (Fig 7).

## Discussion

Habitat loss and fragmentation represent severe threats to jaguars since they exist at low densities, and have large spatial requirements and dispersal patterns mediated by the occurrence of habitat and prey (e.g., [4, 8]). Nonetheless, knowledge of long-range jaguar movement and consequently gene flow remains limited (e.g., [63, 64, 65]). Direct assessments of jaguar movement and dispersal through tracking of VHF- or GPS-collared individuals within Mesoamerican are rare [66–69]. As an alternative, our study applied noninvasive genetic sampling methods to monitor wild Mesoamerican jaguars, with the main objective of assessing their regional conservation genetic status, including genetic diversity and connectivity.

### Genetic diversity in Mesoamerican jaguars

Genetic diversity has important ecological consequences in natural populations, including the maintenance of evolutionary potential and individual fitness to respond to threats such as environmental change and disease (e.g., [9, 70]). All regions sampled within Mesoamerica showed moderate levels of genetic variation (Table 2 and S1 Table), which is in agreement with earlier studies [21, 71]. The discrepancy between the moderately high diversity levels observed and recent demographic declines and the detection of a north-to-south gradient of increasing genetic diversity in Mesoamerican jaguars indicate that current genetic diversity patterns are shaped by both historical (e.g., post-glacial recolonization, historically large population sizes) and contemporary (e.g., restricted gene flow due to habitat loss and fragmentation) events. Diversity levels were lowest for jaguars in northeastern Mexico (Sierra Madre Oriental), representing marginal populations across the species’ range, which are more likely to experience founder events, population bottlenecks, and sporadic changes in population sizes [72]. We also detected moderate levels of inbreeding in Sierra Madre Oriental jaguars, which could have been caused by genetic drift and/or homozygote excess in this potentially small, reproductively isolated population.

Honduran jaguars exhibited the second lowest genetic diversity estimate (Table 2 and S1 Table) among all Mesoamerican sites surveyed. In northern and central Honduras jaguars inhabit fragmented forest patches and are potentially reduced to small population sizes, whereas jaguars living in southeastern Honduras across the Corazón Biosphere are relatively widely distributed with stable population numbers and frequent dispersal events [73–75]. Jaguars studied in Costa Rica, central Belize, and northern Guatemala showed the highest levels of genetic diversity within Mesoamerican sites surveyed and no evidence of heterozygote deficiency, indicating overall moderate to high local population sizes. Jaguars sampled in northern Guatemala and adjoining areas of central Belize are mostly part of the transboundary Selva Maya (The Mayan Forest), which is considered the jaguar’s main stronghold in Central America, and represents one of the largest blocks of contiguous tropical forest left in Mesoamerica (e.g., [13, 65]).

### Genetic structure and contemporary gene flow

Effective conservation and management of threatened carnivore species occurring across complex, heterogeneous landscapes require an assessment of genetic connectivity in natural populations. Patterns of genetic structure in wide-ranging carnivores such as jaguars are shaped by a multitude of contemporary and historical factors, which are of ecological, environmental or anthropogenic nature (e.g., [76, 77–79]). Most importantly, wide-ranging jaguars, naturally preferring well-preserved closed-canopy forest habitat in proximity to riparian areas (e.g., [63]), have a tendency to avoid areas of high human impact (e.g., [80, 81–83]), which are expanding across Mesoamerica. This study revealed important findings regarding genetic connectivity of Mesoamerican jaguars, confirming moderate levels of genetic differentiation and ultimately refuting the hypothesis of contemporary panmixia.

Despite differences in the assumptions of analytical methodologies used here to assess genetic connectivity in Mesoamerican jaguars, individual- and population-based approaches yielded results that were broadly concordant, with the exception of AMOVA. AMOVA supported the inference that Mesoamerican jaguars are a single, admixed population with low genetic differentiation associated among geographic divisions of hypothesized a priori groupings (historically defined subspecies, JCUs, clusters identified by STRUCTURE) (Table 5). Since AMOVA measures gene flow over evolutionary time scales, its results may reflect high levels of historical gene flow or relatively recent demographic contractions in Mesoamerican jaguars, which due to insufficient time did not develop regional genetic divergence. In contrast, hierarchical Bayesian clustering analysis assessing contemporary gene flow at large and fine spatial scales, grouped Mesoamerican jaguars roughly into four major genetic clusters (Figs 2 and 4). However, some of the genetic clusters were not completely isolated with varying levels of admixture, and part of the genetic differentiation detected could be explained through simple isolation-by-distance patterns (Fig 6). The principal component analysis and spatial autocorrelation analysis corroborated the presence of genetic substructuring (Figs 5 and 7). Spatial autocorrelation analysis approximated the extent of positive genetic structure (‘genetic neighborhood’) at ~ 340km (Fig 7), suggesting that jaguar movements beyond this distance are geographically restricted and nonrandom.

Genetic subdivision among Mesoamerican sites included in this study was most pronounced between jaguars sampled at the Sierra Madre Oriental in northeastern Mexico and Selva Maya (Fig 4A), and Selva Maya and Honduras (Fig 4B). This was not surprising because these regions are either geographically isolated or separated by large areas of human-dominated landscape. For instance, Sierra Madre Oriental jaguars occur in geographically isolated and small forest patches surrounded by human-altered landscapes, including cattle pastures and secondary vegetation (e.g., [84, 85]). Previous studies have identified the need to promote connectivity of jaguar populations within this region [86–88]. Dueñas-López, Rosas [89] assessed habitat connectivity for Sierra Madre Oriental jaguars, and classified 48% of the landscape as high-resistance for jaguar movement, while also identifying stepping stone habitat and several dispersal routes with potential for southward jaguar movement to Oaxaca state. However, our pair-wise *F*_*ST*_ and D_est_ estimates (Table 4), PCA (Fig 5), and hierarchical STRUCTURE analysis (Fig 2) suggested that Sierra Madre Oriental jaguars showed unique genetic signatures in comparison to other Mesoamerican sites included in this study. Nonetheless, sample sizes were low and need to be interpreted carefully. We also found a positive correlation between geographic distance and genetic differentiation for Sierra Madre Oriental and Selva Maya jaguars (Fig 6B), indicating that the genetic subdivision in this data set could be an artifact of the underlying IBD pattern and thus geographic distance among these sampling sites may be partially driving the genetic divergence detected. Recently, Zanin, Adrados [90] also reported beginning genetic divergence for jaguars studied in northwestern Mexico from southern parts of the country, which represents another region of conservation concern regarding genetic connectivity within Mexico. Our genetic analysis also identified a potential first-generation migrant (i.e. disperser) by genetically assigning the jaguar sampled outside of Totonpec Villa de Morelos in the Sierra Mixe district of Oaxaca state to the Selva Maya region (Table 3), indicating genetic connectivity between southern Mexico and northern Guatemala. However, it is important to note here that the assignment of first-generation migrants to different localities may be biased due to our clustered sampling design and incomplete range coverage. Given the small number of jaguar samples included for Mexico, these results need to be corroborated using more comprehensive genetic surveys.

Jaguars studied across sampling sites in central Belize and northern Guatemala, which are part of or located in close proximity to Selva Maya, formed one genetic cluster using Bayesian clustering methods (Fig 4D) and PCA (Fig 5), indicating moderate to high levels of contemporary gene flow among these localities. Belizean jaguars included in this study were sampled within the Maya Mountain Massif in south-central Belize (Cockscomb Basin Wildlife Sanctuary) and within or in close proximity to the Central Belize Corridor, which provides the last remaining connection for dispersing jaguars between the northern forest block and the Maya Mountain Massif in Belize. Based on our analysis, we confirmed high gene flow among our Selva Maya jaguars in Belize and Guatemala, but it is unclear if jaguars located in central- and south-central Belize disperse to Guatemala and back through the Central Belize Corridor and the northern forest block or if they more commonly cross over through the Maya Mountain Massif. A previous conservation genetics study on Belizean jaguars [23], which also included DNA samples from several other regions (north, north-central, central, south-central, south) within Belize, reported moderate genetic subdivision of Belizean jaguars into a northern and southern genetic cluster. These findings corresponded largely to patterns of habitat fragmentation and human disturbance in Belize, indicating that rapid human population growth, land conversion, and expansion of agricultural areas across central Belize subtlety affect movements in jaguars, even on a fine-spatial scale and in areas with large tracts of forest such as the Selva Maya nearby. The study emphasized the importance of maintaining and increasing genetic connectivity of jaguars on fine-spatial scales through strengthening formerly identified movement corridors (e.g., Central Belize Corridor) [23].

Genetic differentiation between Selva Maya and Honduran jaguars appeared to be more substantial than expected considering their close geographic proximity. Bayesian clustering (Figs 2 and 4B) and PCA (Fig 5) analyses indicated that jaguars sampled at protected areas in northern (Jeanette Kawas National Park, Pico Bonito National Park) and southeastern (Río Plátano Biosphere Reserve) Honduras are genetically differentiated from Selva Maya jaguars by forming their own genetic cluster with high individual membership probabilities. This suggested potentially low levels of gene flow and genetic admixture among Selva Maya and Honduran jaguars. Our results confirmed findings by two earlier studies [18, 91], which predicted that habitat connectivity for jaguars across this region may be limited. Zeller, Rabinowitz [18] was unable to identify movement corridors for jaguars between the Sierra de las Minas JCU in southern Guatemala and the Pico Bonito/Texiguat JCU in north-central Honduras using least-cost path corridor analysis. Calderon Quinonez [91] delimited a semi-continuous movement corridor in eastern Guatemala that connected with jaguar corridors in southern Belize and western Honduras, and stated that only one-third of the potential Guatemala-Honduras connection is functional for jaguar movement. In addition, Honduras has been facing rapid land cover changes and high deforestation rates, which have severely increased over the last two decades and are now among the highest in Latin America and the world [92], putting remaining stepping stone habitat for jaguars across this region at further risk. The evidence of limited gene flow detected here is particularly concerning since this region represents a critical link for jaguars dispersing through the Mesoamerican Biological Corridor, connecting two areas of increased conservation importance: (1) the tri-national Selva Maya spanning across protected areas in southern Mexico, northern Guatemala, and central Belize, and (2) the Río Plátano Biosphere Reserve in southern Honduras, which is part of La Moskitia (the largest wilderness area in Central America), and the transboundary Corazón Biosphere JCU (~ 21,659 km^2^). Based on these results we declare this region of high conservation concern and recognize the need to conduct further genetic sampling for jaguars across eastern, central, southern Guatemala and most of Honduras to genetically ground-truth functional connectivity of previously identified movement corridors.

Interestingly, we detected a potential first-generation migrant (i.e. disperser) at the Río Plátano Biosphere Reserve in southern Honduras (Table 3), which was genetically assigned to Costa Rica. The Bayesian clustering analysis also suggested that Honduran jaguars exhibited moderate levels of genetic admixture with Costa Rican jaguars (Figs 2 and 4C). However, it is unclear how reliable these results are since our analysis did not include genetic data from jaguars in Nicaragua. Nicaragua, which retains large portions of its native forest cover in the eastern part of the country, has two JCUs (Corazón Biosphere, Cerro Silva-Indio Maíz-Tortuguero) connected by the longest movement corridor identified for jaguars within Central America (Bosawas-Wawashan and Wawashan-Cerro Silva Corridors) [75]. Large-scale movement of jaguars along the corridor has not yet been studied, but Petracca, Hernández-Potosme [82] identified two areas of conservation concern within the corridor, and stated that agricultural encroachment limited the presence of jaguars and several larger prey species at these sites. Jaguar movement and resultant gene flow through Nicaragua is additionally threatened by the construction of a navagable canal and other associated infrastructures (e.g., highways) connecting the Atlantic and Pacific Oceans in southern Nicaragua (e.g., [16, 93]). A recent study [16] stated that the proposed canal zone currently has low occupancy for jaguars and other large mammals, and that the completion of this project has high potential to extirpate jaguars and other species from this area, which would most likely also have ramnifications for jaguars beyond the canal zone.

For Costa Rica, the Bayesian clustering analysis included DNA samples obtained across northern, central, and southern Costa Rica, suggested no genetic subdivision of Costa Rican jaguars (Figs 2 and 4E). Costa Rica has an extensive protected area system (26% of the country) [94], and our results implied that genetic connectivity levels for Costa Rican jaguars are still relatively high. A previous genetic study on Costa Rican jaguars identified fine-scale genetic structure within Costa Rica and described jaguars sampled at northeastern Tortuguero National Park as genetically differentiated from the remaining sampling sites [29]. Due to increasing levels of human impact (e.g., land conversion, human-wildlife conflict) across Costa Rica, we recommend expanding current genetic monitoring efforts to include areas previously not surveyed (e.g., Talamanca). In addition, expanding genetic monitoring efforts across Costa Rica’s neighboring countries of Nicaragua and Panama would be highly valuable and create a more complete picture of functional connectivity levels across southern Central America.

As recommended by previous empirical studies (e.g., [51]), we employed hierarchical genetic structure analysis to detect cryptic patterns of genetic divergence. However, larger and more even sample sizes for some sites and more continuous sampling in general would be beneficial to confirm moderate levels of genetic subdivision detected for jaguars across Mesoamerica. In addition, we cannot rule out the possibility that patterns of genetic differentiation detected here were biased due to clustered sampling or an uneven sex ratio of jaguars included in the study (e.g., [52, 95]). To minimize these errors, we emphasize the importance of conducting in-depth monitoring efforts at fine- and large- spatial scales, which also include areas currently not represented in this study (e.g., most of Mexico; central and southern Guatemala; Nicaragua; eastern Costa Rica; Panama).

### Implications for conservation and management of Mesoamerican jaguars

An accurate understanding of contemporary genetic connectivity is key to preserve genetic health of wild jaguar populations existing across fragmented landscapes. This study detected moderate to relatively high levels of genetic diversity and low to moderate genetic differentiation in Mesoamerican jaguars at different hierarchical levels. The results presented here have several important implications for conservation and management of wild jaguar populations across Mesoamerica, and elsewhere within their range. First, since jaguars are starting to exhibit genetic differentiation within Mesoamerica, attention should be given to keeping and restoring functional connectivity between core jaguar populations by increasing structural connectivity (e.g., wildlife corridors) and minimizing human disturbance. Our results suggested that genetic connectivity is less certain across central regions of Mesoamerica (Honduras and surrounding sites). More complete genetic sampling is needed to examine other regions of conservation concern (e.g., Mexico, Nicaragua). Second, since habitat loss and fragmentation have been identified as primary global threats to mammals, interdisciplinary research efforts combining large-scale genetic monitoring with landscape genetics methodologies need to be conducted to examine if and how different landscape features, as well as varying levels of structural connectivity, shape patterns of contemporary gene flow in wide-ranging carnivores such as jaguars. Third, while the landscape is of key importance with respect to functional connectivity, ex- and intrinsic factors potentially impacting jaguar movement, including population densities of jaguars, their prey, and other competing wildlife species, need to be examined simultaneously with the genetic monitoring effort. In conclusion, we recommend comprehensive regional conservation and management for threatened species such as jaguars, which integrate interdisciplinary research efforts, including conservation and landscape genetic studies at fine- and large spatial scales into their decision-making.

## Supporting Information

S1 Fig Results of hierarchical STRUCTURE analysis in Mesoamerican jaguars. The optimal number of genetic clusters (*K*) in Mesoamerican jaguars using STRUCTURE, version 2.3.4 [47] was chosen based on posterior probability (mean L(*K*), A) and delta *K* (Δ*K*, mean (|L”(*K*)|)/SD(L(*K*)), D) for each *K* value. Bayesian clustering analysis was conducted for jaguars detected in (a) Guatemala, Belize, and Honduras (*n* = 72), (b) Honduras and Costa Rica (*n* = 43), (c) Guatemala and Belize (*n* = 65), and (d) Costa Rica (*n* = 36). SD, standard deviation; L’(*K*), mean rate of change of the likelihood distribution (B); |L”(*K*)|, absolute value of the 2^nd^ order rate of change of the likelihood distribution (C).
(DOCX)

S2 Fig Genetic structure in Mesoamerican jaguars excluding closely related individuals. Results were obtained from Bayesian clustering analysis (*K* = 2) using STRUCTURE, version 2.3.4 [47] and genotype data from 12 microsatellite loci for jaguars (*n* = 48), excluding closely related individuals. Individuals were collected at various sampling sites (1–3, northern Guatemala; 4–27, central Belize; 28–30, Honduras; 31–48, Costa Rica). (a) STRUCTURE barplot—vertical bars represent individuals and the color of each bar visualizes the % of membership (*Q*) the individual belongs to the genetic clusters (*K*) identified. (b) The optimal number of genetic clusters (*K*) in was chosen based on posterior probabilities (mean L(*K*), A) and delta *K* (Δ*K*, mean (|L”(*K*)|)/SD(L(*K*)), D) for each *K* value. SD, standard deviation; L’(*K*), mean rate of change of the likelihood distribution (B); |L”(*K*)|, absolute value of the 2^nd^ order rate of change of the likelihood distribution (C).
(DOCX)

S1 Materials and Methods PCR reactions and thermocycling conditions for multiplex 1–5. (DOCX)

S1 Table Summary statistics of genetic diversity by locus for Mesoamerican jaguars. Estimates of genetic diversity for jaguars (*n* = 115) detected across five Mesoamerican countries (Belize, Costa Rica, Guatemala, Honduras, Mexico), including number of alleles (*N*_*A*_), rarified allelic richness (*A*_*R*_), expected heterozygosity (*H*_*E*_), and inbreeding coefficient (F_IS_)_._ *n*, number of individual felids; *SD*, standard deviation.
(DOCX)

S2 Table Hardy-Weinberg equilibrium and null allele analysis for Mesoamerican jaguars. *P*-value for the Hardy-Weinberg equilibrium (HWE) test (*P*_*HW*_), and frequency of null alleles (*F*_*Null*_) at 12 microsatellite loci for 115 jaguar samples collected across five Mesoamerican countries (Belize, Costa Rica, Guatemala, Honduras, Mexico). *n*, number of individuals.
(DOCX)

## References

[1] Nowell K, Jackson P. Wild cats: status survey and conservation action plan International Union for Conservation of Nature, Gland, Switzerland 1996.

[2] Sanderson EW, Redford KH, Chetkiewicz CLB, Medellin RA, Rabinowitz AR, Robinson JG, et al Planning to save a species: the jaguar as a model. Conserv Biol. 2002;16(1):58–72. 10.1046/j.1523-1739.2002.00352.x 35701976

[3] Seymour KL. Panthera onca. Mammalian Species. 1989;340:1–9.

[4] Sunquist ME, Sunquist F. Wild cats of the world 1^st^ ed. Chicago: University of Chicago Press; 2002.

[5] Macdonald DW, Loveridge AJ. Biology and conservation of wild felids New York: Oxford University Press; 2010.

[6] Rabinowitz A, Zeller KA. A range-wide model of landscape connectivity and conservation for the jaguar, *Panthera onca*. Biol Conserv. 2010;143(4):939–45.

[7] Urquiza-Haas T, Peres CA, Dolman PM. Regional scale effects of human density and forest disturbance on large-bodied vertebrates throughout the Yucatan Peninsula, Mexico. Biol Conserv. 2009;142(1):134–48.

[8] Crooks KR. Relative Sensitivities of Mammalian Carnivores to Habitat Fragmentation. Conserv Biol. 2002;16(2):488–502.

[9] Allendorf FW, Luikart G, Aitken SN. Conservation and the genetics of populations 2^nd^ ed. Oxford, United Kingdom: Wiley-Blackwell; 2013.

[10] Lacy R. Importance of genetic variation to the viability of Mammalian populations. J Mammal. 1997;78(2):320–35.

[11] Frankham R. Genetics and extinction. Biol Conserv. 2005;126(2):131–40.

[12] Caso A, Lopez-Gonzalez C, Payan E, Eizirik E, de Oliviera T, Leite-Pitman R, Kelly M, Valderrama C. Panthera onca The IUCN Red List of Threatened Species 2008: IUCN Species Survival Commission Gland, Switzerland; 2008 Available: http://www.iucnredlist.org/details/15953/0

[13] Swank WG, Teer J. Status of the jaguar—1987. Oryx. 1989;23:14–21.

[14] Redo DJ, Grau HR, Aide TM, Clark ML. Assymetric forest transition driven by the interaction of socioeconomic development and environmental heterogeneity in Central America. Proceedings of the National Academy of Sciences. 2012;109(23):8839–44. 10.1073/pnas.1201664109 PMC3384153 22615408

[15] Harvey CA, Komar O, Chazdon R, Ferguson BG, Finegan B, Griffith DM, Martinez-Ramos M, Morales H, Nigh R, Soto-Pinto L. Integrating agricultural landscapes with biodiversity conservation in the Mesoamerican hotspot. Conservation Biology. 2008;22(1):8–15. 10.1111/j.1523-1739.2007.00863.x 18254848

[16] Jordan CA, Schank CJ, Urquhart GR, Dans AJ. Terrestrial Mammal Occupancy in the Context of Widespread Forest Loss and a Proposed Interoceanic Canal in Nicaragua's Decreasingly Remote South Caribbean Region. PLoS ONE. 2016;11(3):e0151372 10.1371/journal.pone.0151372 27007122 PMC4805289

[17] DeClerck FAJ, Chazdon R, Holl KD, Milder JC, Finegan B, Martinez-Salinas A, et al Biodiversity conservation in human-modified landscapes of Mesoamerica: Past, present and future. Biol Conserv. 2010;143(10):2301–13.

[18] Zeller KA, Rabinowitz A, Salon-Perez R, Quigley H. The Jaguar Corridor Initiative: A Range-Wide Conservation Strategy In: Ruiz-García M, Shostell J, editors. Molecular population genetics, evolutionary biology and biological conservation of Neotropical carnivores. New York: Nova Science Publishers, Inc.; 2013 pp. 629–59.

[19] Zanin M, Palomares F, Brito D. What we (don’t) know about the effects of habitat loss and fragmentation on felids. Oryx. 2015;49(1):96–106.

[20] Schwartz MK, Luikart G, Waples RS. Genetic monitoring as a promising tool for conservation and management. Trends Ecol Evol. 2007;22(1):25–33. 10.1016/j.tree.2006.08.009 16962204

[21] Eizirik E, Kim JH, Menotti-Raymond M, Crawshaw PG, O'Brien SJ, Johnson WE. Phylogeography, population history and conservation genetics of jaguars (*Panthera onca*, Mammalia, Felidae). Mol Ecol. 2001;10(1):65–79. 11251788 10.1046/j.1365-294x.2001.01144.x

[22] Ruiz-García M, Vásquez C, Murillo A, Pinedo-Castro M, Alvarez D. Population genetics and phylogeography of the largest wild cat in the Americas: an analysis of the jaguar by means of microsatellites and mitochondrial gene sequences In: Ruiz-García M, Shostell J, editors. Molecular population genetics, evolutionary biology and biological conservation of Neotropical carnivores. New York: Nova Science Publishers, Inc.; 2013 pp. 413–64.

[23] Wultsch C, Waits LP, Kelly MJ. A comparative analysis of genetic diversity and structure in jaguars (*Panthera onca*), pumas (*Puma concolor*), and ocelots (*Leopardus pardalis*) in fragmented landscapes of a critical Mesoamerican linkage zone. PLoS ONE. 2016;11(3):e0151043 10.1371/journal.pone.0151043 26974968 PMC4790928

[24] Haag T, Santos AS, Sana DA, Morato RG, Cullen L, Crawshaw PG, et al The effect of habitat fragmentation on the genetic structure of a top predator: loss of diversity and high differentiation among remnant populations of Atlantic Forest jaguars (*Panthera onca*). Mol Ecol. 2010;19(22):4906–21. 10.1111/j.1365-294X.2010.04856.x 21040050

[25] Kelly MJ, Betsch J, Wultsch C, Mesa B, Mills LS. Noninvasive sampling for carnivores In: Boitani L, Powell RA, editors. Carnivore Ecology and Conservation—A Handbook of Techniques. New York: Oxford University Press; 2012 pp. 47–69.

[26] Wultsch C, Waits LP, Hallerman EM, Kelly MJ. Optimizing collection methods for noninvasive genetic sampling of Neotropical felids. Wildlife Society Bulletin. 2015;39(2):403–12.

[27] Pocock RI. The races of jaguar (*Panthera onca*). Novitates Zoologicae. 1939;41:406–22.

[28] Larson SE. Taxonomic Re-Evaluation of the Jaguar. Zoo Biology. 1997;16(2):107–20.

[29] Soto Fournier S. Diversidad genética y estructura poblacional de Panthera onca y Puma concolor (Carnivora: Felidae) en Costa Rica. M.Sc. Thesis, Universidad de Costa Rica. 2014.

[30] Caragiulo A, Dias-Freedman I, Clark J, Rabinowitz S, Amato G. Mitochondrial DNA sequence variation and phylogeography of Neotropic pumas (*Puma concolor*). Mitochondrial DNA. 2014;25:304–12. 10.3109/19401736.2013.800486 23789770

[31] Menotti-Raymond M, O'Brien SJ. Evolutionary conservation of ten microsatellite loci in four species of Felidae. The Journal of Heredity. 1995;86(4):319–22. 7658003 10.1093/oxfordjournals.jhered.a111594

[32] Menotti-Raymond M, David VA, Lyons LA, Schaffer AA, Tomlin JF, Hutton MK, et al A genetic linkage map of microsatellites in the domestic cat (*Felis catus*). Genomics. 1999;57(1):9–23. 10.1006/geno.1999.5743 10191079

[33] Caragiulo A, Kang Y, Rabinowitz S, Dias-Freedman I, Loss S, Zhou X, et al Presence of the Endangered Amur tiger *Panthera tigris altaica* in Jilin Province, China, detected using non-invasive genetic techniques. Oryx. 2015;49(4):632–5.

[34] Wei K, Zhang Z, Zhang W, Xu X, Liang X, He G, et al PCR-CTPP: a rapid and reliable genotyping technique based on ZFX/ZFY alleles for sex identification of tiger (*Panthera tigris*) and four other endangered felids. Conserv Genet. 2008;9(1):225–8.

[35] Pilgrim KL, McKelvey KS, Riddle AE, Schwartz MK. Felid sex identification based on noninvasive genetic samples. Molecular Ecology Notes. 2005;5(1):60–1.

[36] Taberlet P, Griffin S, Goossens B, Questiau S, Manceau V, Escaravage N, et al Reliable genotyping of samples with very low DNA quantities using PCR. Nucleic Acids Res. 1996;24(16):3189–94. 8774899 10.1093/nar/24.16.3189 PMC146079

[37] Mills LS, Citta JJ, Lair KP, Schwartz MK, Tallmon DA. Estimating animal abundance using noninvasive DNA sampling: promise and pitfalls. Ecol Appl. 2000;10(1):283–94.

[38] Waits LP, Luikart G, Taberlet P. Estimating the probability of identity among genotypes in natural populations: cautions and guidelines. Mol Ecol. 2001;10(1):249–56. 11251803 10.1046/j.1365-294x.2001.01185.x

[39] Peakall ROD, Smouse PE. GenAlEx 6: genetic analysis in Excel. Population genetic software for teaching and research. Molecular Ecology Notes. 2006;6(1):288–95. 10.1093/bioinformatics/bts460 PMC3463245 22820204

[40] Lonsinger RC, Waits LP. ConGenR: rapid determination of consensus genotypes and estimates of genotyping errors from replicated genetic samples. Conserv Genet Resour. 2015;7(4):841–3.

[41] R Development Core Team. R: a language and environment for statistical computing R Foundation for Statistical Computing, Vienna, Austria; 2011.

[42] Keenan K, McGinnity P, Cross TF, Crozier WW, Prodöhl PA. *diveRsity*: An R package for the estimation and exploration of population genetics parameters and their associated errors. Methods Ecol Evol. 2013;4(8):782–8.

[43] Goudet J. *Hierfstat*, a package for R to compute and test hierarchical *F*‐statistics. Molecular Ecology Notes. 2005;5(1):184–6.

[44] Raymond M, Rousset F. GENEPOP—Population-genetics software for exact tests and ecumenicism. J Hered. 1995;86(3):248–9.

[45] Van Oosterhout C, Hutchinson WF, Wills DPM, Shipley P. MICRO-CHECKER: software for identifying and correcting genotyping errors in microsatellite data. Molecular Ecology Notes. 2004;4(3):535–8.

[46] Rice WR. Analyzing Tables of Statistical Tests. Evolution. 1989;43(1):223–5. 28568501 10.1111/j.1558-5646.1989.tb04220.x

[47] Pritchard JK, Stephens M, Donnelly P. Inference of Population Structure Using Multilocus Genotype Data. Genetics. 2000;155(2):945–59. 10835412 10.1093/genetics/155.2.945 PMC1461096

[48] Hubisz MJ, Falush D, Stephens M, Pritchard JK. Inferring weak population structure with the assistance of sample group information. Mol Ecol Resour. 2009;9(5):1322–32. 10.1111/j.1755-0998.2009.02591.x 21564903 PMC3518025

[49] Evanno G, Regnaut S, Goudet J. Detecting the number of clusters of individuals using the software STRUCTURE: a simulation study. Mol Ecol. 2005;14(8):2611–20. 10.1111/j.1365-294X.2005.02553.x 15969739

[50] Francis RM. Pophelper: an R package and web app to analyse and visualize population structure. Mol Ecol Resour. 2016 10.1111/1755-0998.12509 26850166

[51] Balkenhol N, Holbrook JD, Onorato D, Zager P, White C, Waits LP. A multi-method approach for analyzing hierarchical genetic structures: a case study with cougars *Puma concolor*. Ecography. 2014;37:552–63.

[52] Tucker J, Schwartz MK, Truex R, Wisely S, Allendorf FW. Sampling affects the detection of genetic subdivision and conservation implications for fisher in the Sierra Nevada. Conserv Genet. 2014;15:123–36.

[53] Kalinowski ST, Wagner AP, Taper ML. ML-RELATE: a computer program for maximum likelihood estimation of relatedness and relationship. Molecular Ecology Notes. 2006;6(2):576–9.

[54] Jombart T. *adegenet*: a R package for the multivariate analysis of genetic markers. Bioinformatics. 2008;24(11):1403–5. 10.1093/bioinformatics/btn129 18397895

[55] Piry S, Alapetite A, Cornuet JM, Paetkau D, Baudouin L, Estoup A. GENECLASS2: A software for genetic assignment and first-generation migrant detection. J Hered. 2004;95(6):536–9. 10.1093/jhered/esh074 15475402

[56] Rannala B, Mountain JL. Detecting immigration by using multilocus genotypes. Proc Natl Acad Sciences. 1997;94(17):9197–9201. 10.1073/pnas.94.17.9197 PMC23111 9256459

[57] Paetkau D, Slade R, Burden M, Estoup A. Genetic assignment methods for the direct, real-time estimation of migration rate: a simulation-based exploration of accuracy and power. Mol Ecol. 2004;13(1):55–65. 14653788 10.1046/j.1365-294x.2004.02008.x

[58] Weir BS, Cockerham CC. Estimating *F*-statistics for the analyis of population structure. Evolution. 1984;38(6):1358–70. 28563791 10.1111/j.1558-5646.1984.tb05657.x

[59] Jost L. *G*_*ST*_ and its relatives do not measure differentiation. Mol Ecol. 2008;17(18):4015–26. 19238703 10.1111/j.1365-294x.2008.03887.x

[60] Excoffier L, Smouse PE, Quattro JM. Analysis of molecular variance inferred fom metric distances among DNA haplotypes—Application to human mitochondrial-DNA restriction data. Genetics. 1992;131(2):479–91. 1644282 10.1093/genetics/131.2.479 PMC1205020

[61] Dray S, Dufour A-B. The *ade4* package: implementing the duality diagram for ecologists. Journal of statistical software. 2007;22(4):1–20.

[62] Goslee SC, Urban DL. The *ecodist* package for dissimilarity-based analysis of ecological data. Journal of Statistical Software. 2007;22(7):1–19.

[63] Crawshaw PG, Quigley HB. Jaguar spacing, activity and habitat use in a seasonally flooded environment in Brazil. J Zool. 1991;223(3):357–70.

[64] Schaller GB, Crawshaw PG. Movement patterns of jaguar. Biotropica. 1980;12(3):161–8.

[65] Ceballos G, Chavez C, Zarza H, Manterola C. Ecología y conservación del jaguar en la región de Calakmul. Biodiversitas. 2005;62:1–7.

[66] Rabinowitz AR, Nottingham BG. Ecology and behavior of the jaguar (*Panthera onca*) in Belize, Central America. J Zool. 1986;210(1):149–59.

[67] Colchero F, Conde DA, Manterola C, Chavez C, Rivera A, Ceballos G. Jaguars on the move: modeling movement to mitigate fragmentation from road expansion in the Mayan Forest. Anim Conserv. 2011;14(2):158–66.

[68] Conde DA, Colchero F, Zarza H, Christensen NL, Sexton JO, Manterola C, et al Sex matters: Modeling male and female habitat differences for jaguar conservation. Biol Conserv. 2010;143(9):1980–8.

[69] Figueroa OA. The ecology and conservation of jaguars (Panthera onca) in central Belize: conservation status, diet, movement patterns and habitat use. Ph.D. Thesis, University of Florida. 2013. Available: http://ufdc.ufl.edu/UFE0045191/00001

[70] Hughes AR, Inouye BD, Johnson MTJ, Underwood N, Vellend M. Ecological consequences of genetic diversity. Ecol Lett. 2008;11(6):609–23. 10.1111/j.1461-0248.2008.01179.x 18400018

[71] Wultsch C, Waits LP, Kelly MJ. Noninvasive individual and species identification of jaguars (*Panthera onca*), pumas (*Puma concolor*), and ocelots (*Leopardus pardalis*) in Belize, Central America using cross-species microsatellites and faecal DNA. Molecular Ecology Resources. 2014;14(6):1171–82. 10.1111/1755-0998.12266 24751217

[72] Eckert CG, Samis KE, Lougheed SC. Genetic variation across species’ geographical ranges: the central–marginal hypothesis and beyond. Mol Ecol. 2008;17(5):1170–88. 10.1111/j.1365-294X.2007.03659.x 18302683

[73] Castañeda F, Herrera L, Pereira S, D. S. Estado del jaguar (*Panthera onca*) en el Parque Nacional Jeannette Kawas, Honduras. Panthera, New York 2011.

[74] Castañeda F, Pereita S, Solís M. In the middle of the corridor: status of *Panthera onca* at Pico Bonito National Park, Honduras. Rev Mesoamericana. 2011;15(2):73.

[75] Zeller KA. Jaguars in the New Millennium Data Set Update: The State of the Jaguar in 2006. Wildlife Conservation Society, New York 2007.

[76] Rueness EK, Jorde PE, Hellborg L, Stenseth NC, Ellegren H, Jakobsen KS. Cryptic population structure in a large, mobile mammalian predator: the Scandinavian lynx. Mol Ecol. 2003;12(10):2623–33. 12969466 10.1046/j.1365-294x.2003.01952.x

[77] Carmichael LE, Nagy JA, Larter NC, Strobeck C. Prey specialization may influence patterns of gene flow in wolves of the Canadian Northwest. Mol Ecol. 2001;10(12):2787–98. 11903892 10.1046/j.0962-1083.2001.01408.x

[78] Pilot M, Jedrzejewski W, Branicki W, Sidorovich VE, Jedrzejewska B, Stachura K, et al Ecological factors influence population genetic structure of European grey wolves. Mol Ecol. 2006;15(14):4533–53. 10.1111/j.1365-294X.2006.03110.x 17107481

[79] Scognamillo D, Maxit IE, Sunquist M, Polisar J. Coexistence of jaguar (*Panthera onca*) and puma (*Puma concolor*) in a mosaic landscape in the Venezuelan llanos. J Zool. 2003;259(3):269–79.

[80] Michalski F, Peres CA. Anthropogenic determinants of primate and carnivore local extinctions in a fragmented forest landscape of southern Amazonia. Biol Conserv. 2005;124(3):383–96.

[81] Zeilhofer P, Cezar A, Tôrres NM, de Almeida Jácomo AT, Silveira L. Jaguar *Panthera onca* Habitat Modeling in Landscapes Facing High Land-use Transformation Pressure -Findings from Mato Grosso, Brazil. Biotropica. 2014;46(1):98–105.

[82] Petracca LS, Hernández-Potosme S, Obando-Sampson L, Salom-Pérez R, Quigley H, Robinson HS. Agricultural encroachment and lack of enforcement threaten connectivity of range-wide jaguar (*Panthera onca*) corridor. J Nat Conserv. 2014;22(5):436–44.

[83] Colchero F, Conde DA, Manterola C, Chávez C, Rivera A, Ceballos G. Jaguars on the move: modeling movement to mitigate fragmentation from road expansion in the Mayan Forest. Anim Conserv. 2011;14(2):158–66.

[84] Hernández-SaintMartín AD, Rosas-Rosas OC, Palacio-Núñez J, Tarango-Arambula LA, Clemente-Sánchez F, Hoogesteijn AL. Food Habits of Jaguar and Puma in a Protected Area and Adjacent Fragmented Landscape of Northeastern Mexico. Nat Areas J. 2015;35(2):308–17.

[85] Arriaga L, Espinoza JM, Aguilar C, Martínez E, Gómez L, Loa E. Regiones Terrestres Prioritarias de México. México D.F., México: Comisión Nacional para el Conocimiento y uso de la Biodiversidad 2000.

[86] Grigione MM, Menke K, López-González C, List R, Banda A, Carrera J, et al Identifying potential conservation areas for felids in the USA and Mexico: integrating reliable knowledge across an international border. Oryx. 2009;43(1):78.

[87] Villordo-Galván JA, Rosas-Rosas OC, Clemente-Sánchez F, Martínez-Montoya JF, Tarango-Arámbula LA, Mendoza-Martínez G, et al The jaguar (*Panthera onca*) in San Luis Potosí, México. The Southwestern Naturalist. 2010;55(3):394–402.

[88] Rodríguez-Soto C, Monroy-Vilchis O, Maiorano L, Boitani L, Faller JC, Briones MÁ, et al Predicting potential distribution of the jaguar (*Panthera onca*) in Mexico: identification of priority areas for conservation. Diversity and Distributions. 2011;17(2):350–61.

[89] Dueñas-López G, Rosas OCR, Chapa-Vargas L, Bender LC, Tarango-Arámbula LA, Martínez-Montoya JF, et al Connectivity among jaguar populations in the Sierra Madre Oriental, México. Therya. 2015;6(2):449–68.

[90] Zanin M, Adrados B, González N, Roques S, Brito D, Chávez C, et al Gene flow and genetic structure of the puma and jaguar in Mexico. Eur J Wildl Res. 2016;62(4):461–9.

[91] Calderon Quinonez AP. Assessment of movement corridors for jaguars in eastern Guatemala. M.Sc. Thesis, State University of New York. 2013. Available: http://gradworks.umi.com/15/49/1549089.html

[92] Food and Agriculture Organization of the United States. Global forest resources assessment 2010: Main report: Food and Agriculture Organization of the United Nations; 2010. Available: http://www.fao.org/docrep/013/i1757e/i1757e.pdf

[93] Huete-Pérez JA, Alvarez PJJ, Schnoor JL, Rittmann BE, Clayton A, Acosta ML, et al Scientists raise alarms about fast tracking of transoceanic canal through Nicaragua. Environmental Science & Technology. 2015;49(7):3989–96. 25730497 10.1021/acs.est.5b00215

[94] González-Maya JF, Víquez-R LR, Belant JL, Ceballos G. Effectiveness of Protected Areas for Representing Species and Populations of Terrestrial Mammals in Costa Rica. PLoS ONE. 2015;10(5):e0124480 10.1371/journal.pone.0124480 25970293 PMC4430271

[95] Schwartz MK, McKelvey KS. Why sampling scheme matters: the effect of sampling scheme on landscape genetic results. Conserv Genet. 2009;10(2):441–52.

