# Supplementary material for: Genetic Diversity and Population Structure of Mesoamerican Jaguars (Panthera onca): Implications for Conservation and Management
Source: PLoS One. 2016 Oct 26;11(10):e0162377. doi: 10.1371/journal.pone.0162377 (PMC5082669; doi:10.1371/journal.pone.0162377)

**Figure S1. Results of hierarchical STRUCTURE analysis in Mesoamerican jaguars**. The optimal number of genetic clusters (*K*) in Mesoamerican jaguars using STRUCTURE, version 2.3.4 [47] was chosen based on posterior probability (mean L(*K*), A) and delta *K* (Δ*K*, mean (|L”(*K*)|)/SD(L(*K*)), D) for each *K* value. Bayesian clustering analysis was conducted for jaguars detected in (a) Guatemala, Belize, and Honduras (*n* = 72), (b) Honduras and Costa Rica (*n* = 43), (c) Guatemala and Belize (*n* = 65), and (d) Costa Rica (*n* = 36). SD, standard deviation; L’(*K*), mean rate of change of the likelihood distribution (B); |L”(*K*)|, absolute value of the 2^nd^ order rate of change of the likelihood distribution (C).

(a)


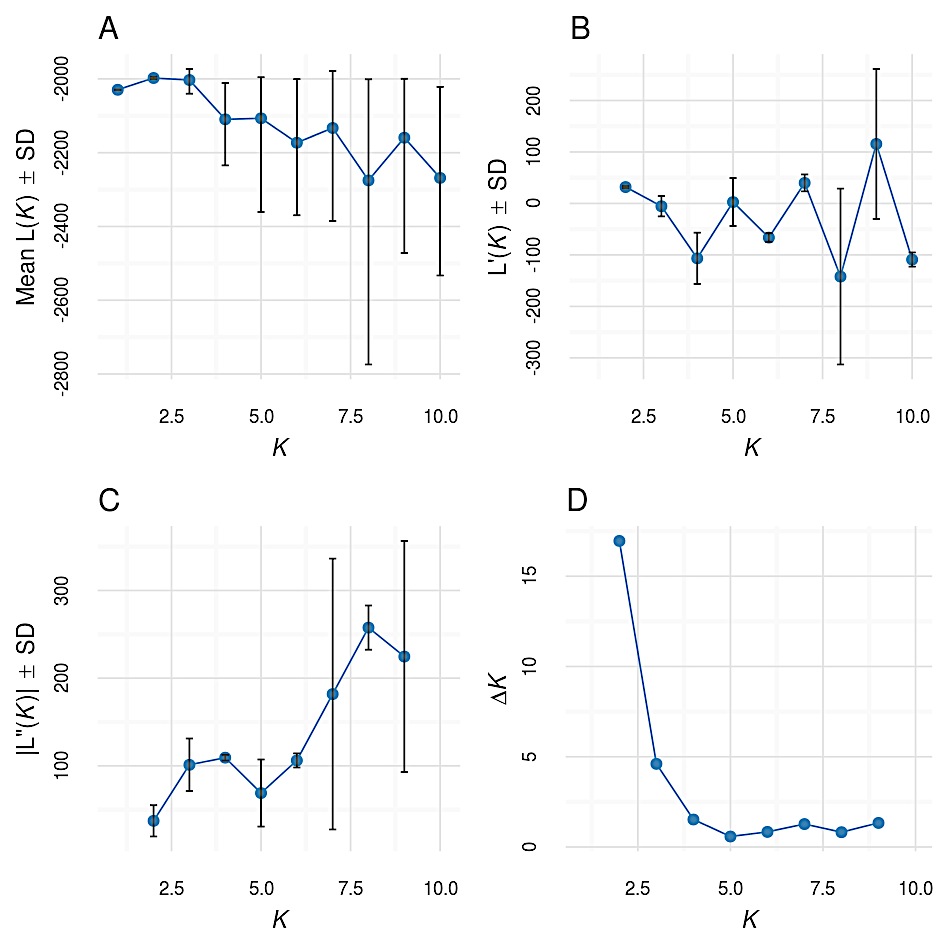


(b)


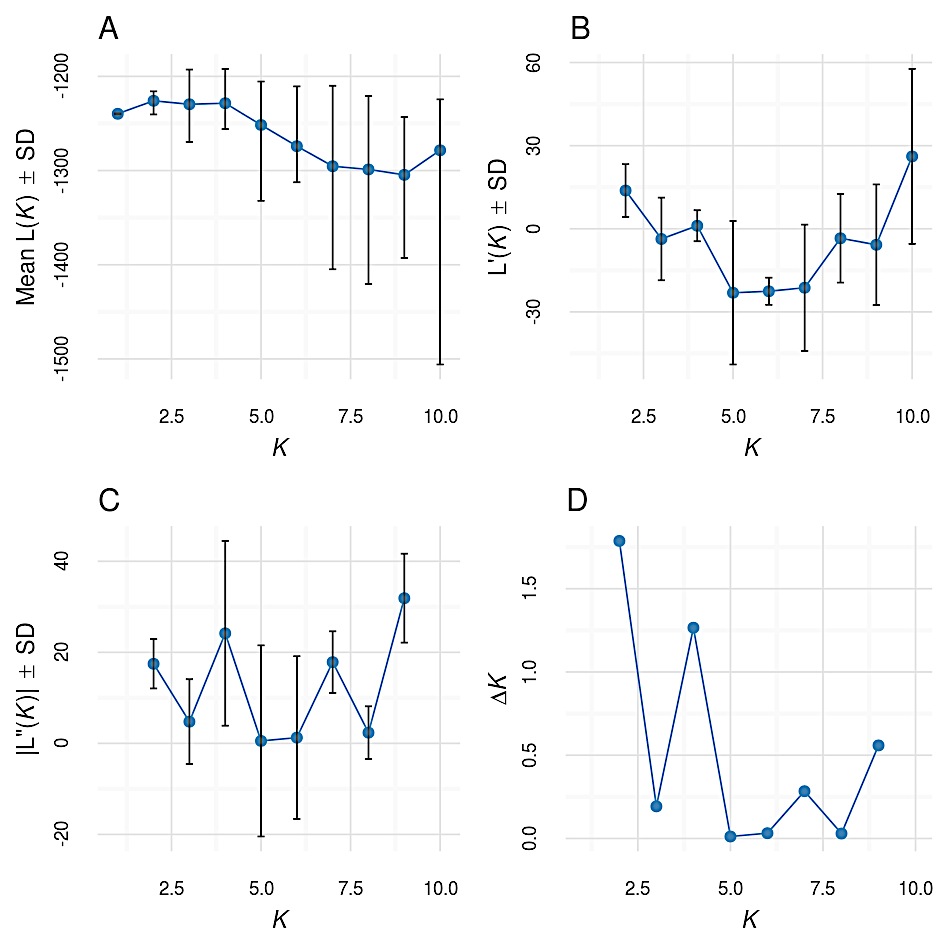


(c)


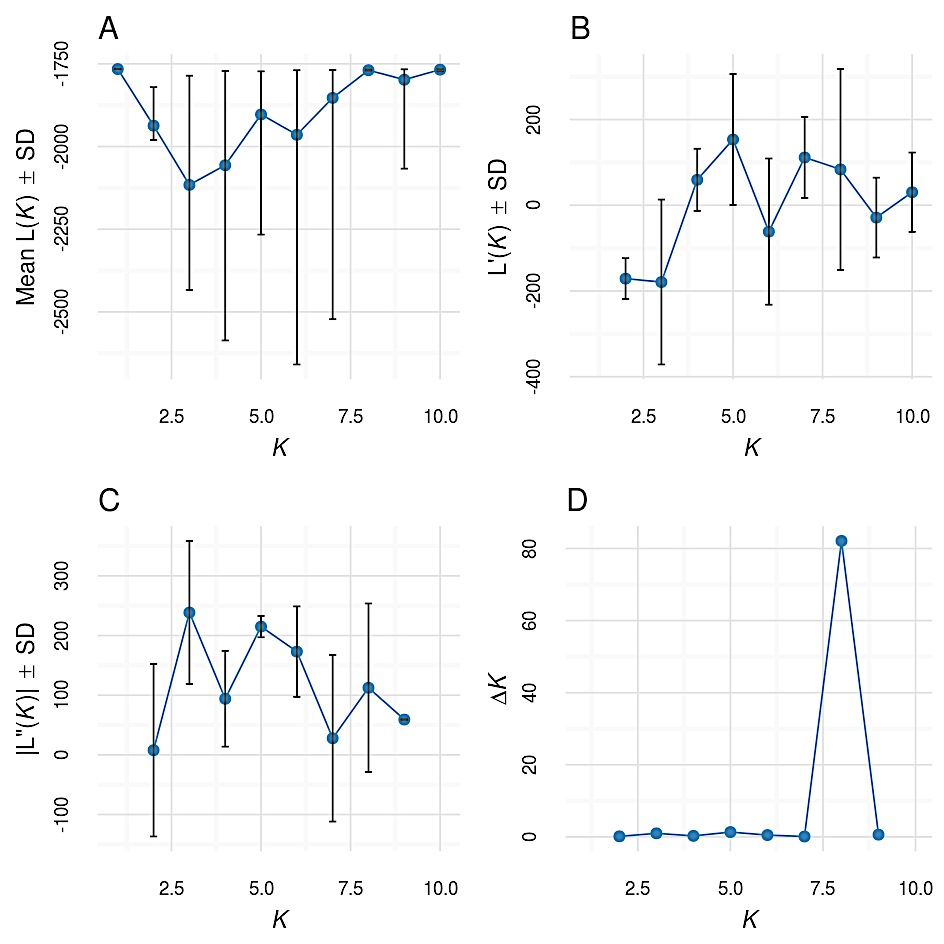


(d)


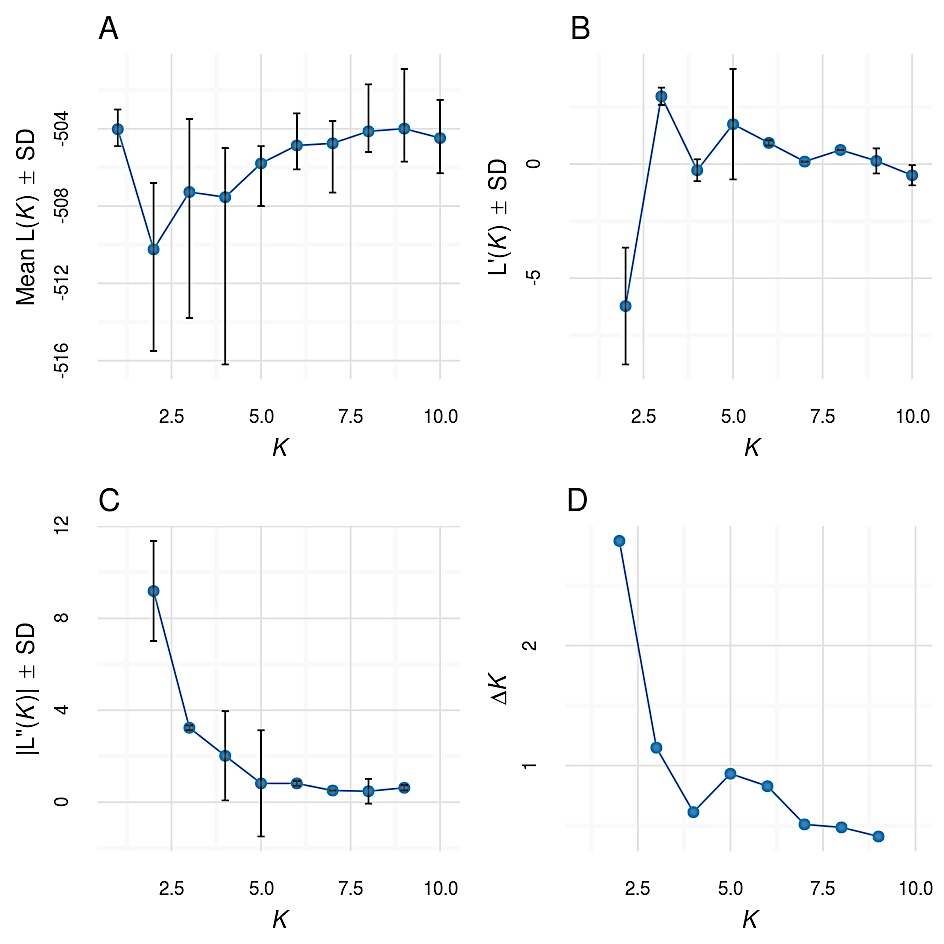

Supplement: S1 Fig — The optimal number of genetic clusters (K) in Mesoamerican jaguars using STRUCTURE, version 2.3.4 [47] was chosen based on posterior probability (mean L(K), A) and delta K (ΔK, mean (|L”(K)|)/SD(L(K)), D) for each K value. Bayesian clustering analysis was conducted for jaguars detected in (a) Guatemala, Belize, and Honduras (n = 72), (b) Honduras and Costa Rica (n = 43), (c) Guatemala and Belize (n = 65), and (d) Costa Rica (n = 36). SD, standard deviation; L’(K), mean rate of change of the likelihood distribution (B); |L”(K)|, absolute value of the 2nd order rate of change of the likelihood distribution (C). (DOCX) [file pone.0162377.s001.docx]
