# Supplementary material for: Genetic Diversity and Population Structure of Mesoamerican Jaguars (Panthera onca): Implications for Conservation and Management
Source: PLoS One. 2016 Oct 26;11(10):e0162377. doi: 10.1371/journal.pone.0162377 (PMC5082669; doi:10.1371/journal.pone.0162377)
Supplement: S2 Fig — Results were obtained from Bayesian clustering analysis (K = 2) using STRUCTURE, version 2.3.4 [47] and genotype data from 12 microsatellite loci for jaguars (n = 48), excluding closely related individuals. Individuals were collected at various sampling sites (1–3, northern Guatemala; 4–27, central Belize; 28–30, Honduras; 31–48, Costa Rica). (a) STRUCTURE barplot—vertical bars represent individuals and the color of each bar visualizes the % of membership (Q) the individual belongs to the genetic clusters (K) identified. (b) The optimal number of genetic clusters (K) in was chosen based on posterior probabilities (mean L(K), A) and delta K (ΔK, mean (|L”(K)|)/SD(L(K)), D) for each K value. SD, standard deviation; L’(K), mean rate of change of the likelihood distribution (B); |L”(K)|, absolute value of the 2nd order rate of change of the likelihood distribution (C). (DOCX) [file pone.0162377.s002.docx]

**Figure S2. Genetic structure in Mesoamerican jaguars excluding closely related individuals.** Results were obtained from Bayesian clustering analysis (*K* = 2) using STRUCTURE, version 2.3.4 [47] and genotype data from 12 microsatellite loci for jaguars (*n* = 48), excluding closely related individuals. Individuals were collected at various sampling sites (1-3, northern Guatemala; 4-27, central Belize; 28-30, Honduras; 31-48, Costa Rica). (a) STRUCTURE barplot - vertical bars **represent individuals and the color of each bar visualizes the % of membership (*Q*) the individual belongs to the genetic clusters (*K*) identified.** **(b)** The optimal number of genetic clusters (*K*) in was chosen based on posterior probabilities (mean L(*K*), A) and delta *K* (Δ*K*, mean (|L”(*K*)|)/SD(L(*K*)), D) for each *K* value. SD, standard deviation; L’(*K*), mean rate of change of the likelihood distribution (B); |L”(*K*)|, absolute value of the 2^nd^ order rate of change of the likelihood distribution (C).

(a)

*Q*


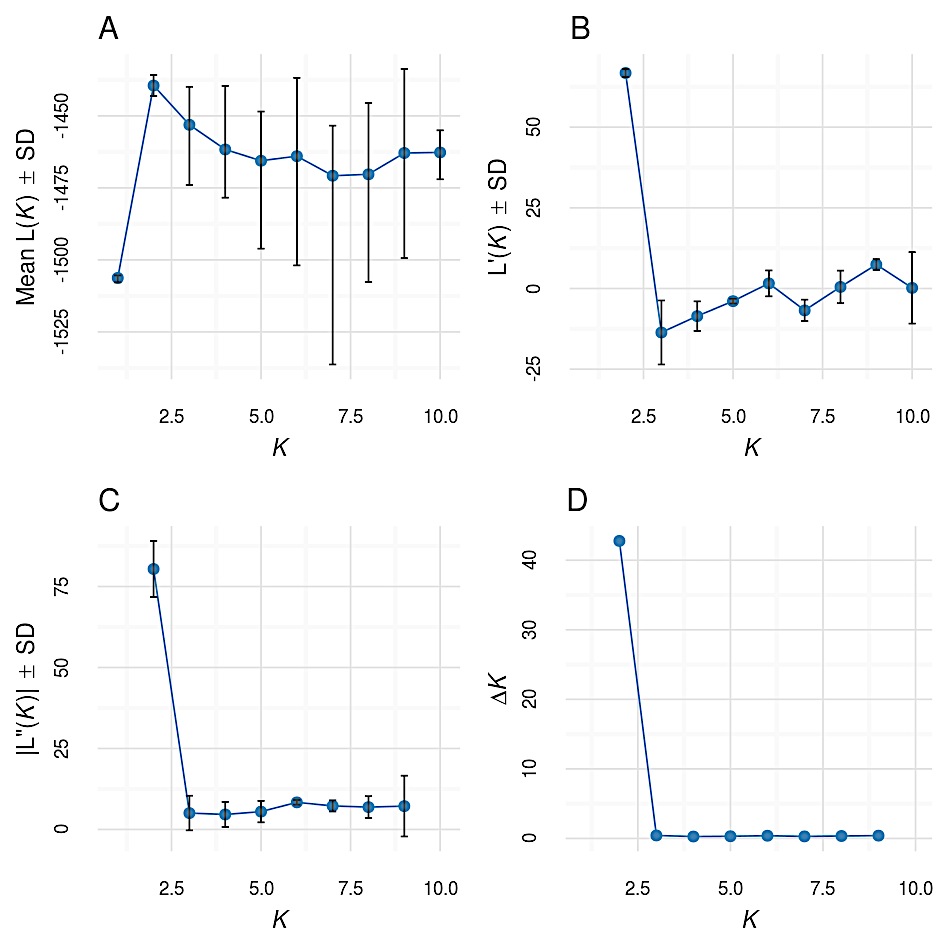
(b)
