# Supplementary material for: Genetic Diversity and Population Structure of Mesoamerican Jaguars (Panthera onca): Implications for Conservation and Management
Source: PLoS One. 2016 Oct 26;11(10):e0162377. doi: 10.1371/journal.pone.0162377 (PMC5082669; doi:10.1371/journal.pone.0162377)
Supplement: S1 Materials and Methods — (DOCX) [file pone.0162377.s003.docx]

**S1 Materials and Methods. PCR reactions and thermocycling conditions for multiplex 1-5.**

Multiplex 1 consisted of 5 µL 1 x concentrated Qiagen Master Mix (Qiagen, Inc., Valencia, CA), 2.25 µL of primers (0.4µM for FCA032, 0.6µM for FCA100, 0.1µM for FCA124), 1.0 µL of 0.5 x concentrated Qiagen Q solution (Qiagen, Inc., Valenica, CA), and 2.0 µL DNA extract. Multiplex 2 consisted of 5 µL 1 x concentrated Qiagen Master Mix, 1.2 µL of primers (0.2µM for FCA126, 0.2µM for FCA212, 0.2µM for FCA229), 1.0 µL of 0.5 x concentrated Qiagen Q solution, 0.8 µL H_2_O, and 2.0 µL DNA extract. Multiplex 3 consisted of 5 µL 1 x concentrated Qiagen Master Mix, 1.2 µL of primers (0.2µM for FCA096, 0.2µM for FCA132, 0.2µM for FCA275), 1.0 µL of 0.5 x concentrated Qiagen Q solution, 0.8 µL H_2_O, and 2.0 µL DNA extract. Multiplex 4 consisted of 5 µL 1 x concentrated Qiagen Master Mix, 2.0 µL of primers (0.2µM for FCA075, 0.8µM for FCA208), 1.0 µL of 0.5 x concentrated Qiagen Q solution, and 2.0 µL DNA extract. Multiplex 5 consisted of 5 µL 1 x concentrated Qiagen Master Mix, 1.6 µL of primers (0.8µM for FCA225), 1.0 µL of 0.5 x concentrated Qiagen Q solution, 0.4 µL H_2_O, and 2.0 µL DNA extract.

Microsatellite PCR amplifications were conducted starting with an initial denaturation step of 15 min at 95 °C; followed by 13 cycles of 30 s at 94 °C for denaturation, 1.5 min at 58.4 °C for multiplex 1, 62.4 °C for multiplex 2, 59.4 °C for multiplex 3, 58.4 °C for multiplex 4, 57.4 °C for multiplex 5 with a decrease in annealing temperature of 0.3 °C in each cycle, and 1 min elongation at 72 °C; followed by 32 cycles of 30 s at 94 °C for denaturation, 1.5 min at 56 °C for multiplex 1, 60 °C for multiplex 2, 57 °C for multiplex 3, 56 °C for multiplex 4, 55 °C for multiplex 5 for annealing, and 1 min elongation at 72 °C; and 30 min at 56 °C for multiplex 1, 60 °C for multiplex 2, 57 °C for multiplex 3, 56 °C for multiplex 4, and 55 °C for multiplex 5 for final elongation.
