# Supplementary material for: Genetic Diversity and Population Structure of Mesoamerican Jaguars (Panthera onca): Implications for Conservation and Management
Source: PLoS One. 2016 Oct 26;11(10):e0162377. doi: 10.1371/journal.pone.0162377 (PMC5082669; doi:10.1371/journal.pone.0162377)
Supplement: S1 Table — Estimates of genetic diversity for jaguars (n = 115) detected across five Mesoamerican countries (Belize, Costa Rica, Guatemala, Honduras, Mexico), including number of alleles (NA), rarified allelic richness (AR), expected heterozygosity (HE), and inbreeding coefficient (FIS). n, number of individual felids; SD, standard deviation. (DOCX) [file pone.0162377.s004.docx]

| **Locus** | **Mexico** (*n* = 7) | | |  | **Guatemala** (*n* = 15) | | | | **Belize** (*n* = 50) | | |  | **Honduras** (*n* = 7) | | |  | **Costa Rica** (*n* = 36) | | |  |
| --- | --- | --- | --- | --- | --- | --- | --- | --- | --- | --- | --- | --- | --- | --- | --- | --- | --- | --- | --- | --- |
|  | ***N_A_*** | ***A_R_*** | ***H_E_*** | ***F_IS_*** | ***N_A_*** | ***A_R_*** | ***H_E_*** | ***F_IS_*** | ***N_A_*** | ***A_R_*** | ***H_E_*** | ***F_IS_*** | ***N_A_*** | ***A_R_*** | ***H_E_*** | ***F_IS_*** | ***N_A_*** | ***A_R_*** | ***H_E_*** | ***F_IS_*** |
| **FCA032** | 3.00 | 2.98 | 0.61 | 0.27 | 5.00 | 4.03 | 0.72 | -0.15 | 5.00 | 3.28 | 0.63 | 0.15 | 5.00 | 4.08 | 0.55 | -0.22 | 6.00 | 3.83 | 0.68 | 0.10 |
| **FCA075** | 4.00 | 3.92 | 0.72 | 0.09 | 7.00 | 5.36 | 0.82 | 0.06 | 10.0 | 5.56 | 0.86 | 0.28 | 3.00 | 2.71 | 0.50 | -0.36 | 7.00 | 4.42 | 0.74 | 0.02 |
| **FCA096** | 4.00 | 3.82 | 0.68 | 0.11 | 6.00 | 3.82 | 0.67 | -0.04 | 6.00 | 3.90 | 0.73 | -0.06 | 4.00 | 3.65 | 0.51 | 0.12 | 7.00 | 4.58 | 0.77 | 0.21 |
| **FCA100** | 3.00 | 2.43 | 0.26 | 0.50 | 4.00 | 3.21 | 0.64 | -0.19 | 5.00 | 2.92 | 0.60 | 0.14 | 2.00 | 2.00 | 0.41 | -0.33 | 5.00 | 2.84 | 0.52 | 0.06 |
| **FCA124** | 3.00 | 2.99 | 0.62 | -0.07 | 5.00 | 3.73 | 0.68 | -0.18 | 4.00 | 3.01 | 0.65 | 0.14 | 6.00 | 4.79 | 0.63 | -0.05 | 6.00 | 3.69 | 0.63 | 0.22 |
| **FCA126** | 2.00 | 1.99 | 0.34 | -0.20 | 3.00 | 2.70 | 0.48 | -0.30 | 6.00 | 4.13 | 0.71 | -0.07 | 3.00 | 2.99 | 0.62 | -0.07 | 4.00 | 3.16 | 0.64 | 0.08 |
| **FCA132** | 2.00 | 2.00 | 0.42 | -0.33 | 2.00 | 1.75 | 0.19 | -0.08 | 2.00 | 1.37 | 0.08 | -0.03 | 4.00 | 3.36 | 0.46 | 0.14 | 6.00 | 3.17 | 0.52 | -0.04 |
| **FCA208** | 4.00 | 4.00 | 0.64 | 0.17 | 6.00 | 3.78 | 0.66 | 0.11 | 7.00 | 4.47 | 0.77 | -0.07 | 6.00 | 5.62 | 0.79 | 0.04 | 11.0 | 5.14 | 0.79 | 0.10 |
| **FCA212** | 2.00 | 2.00 | 0.38 | 0.62 | 2.00 | 2.00 | 0.46 | -0.26 | 2.00 | 1.85 | 0.29 | 0.41 | 2.00 | 2.00 | 0.49 | -0.09 | 3.00 | 2.25 | 0.46 | -0.17 |
| **FCA225** | 5.00 | 5.00 | 0.68 | -0.07 | 3.00 | 2.67 | 0.56 | 0.15 | 4.00 | 3.26 | 0.59 | 0.12 | 4.00 | 3.67 | 0.63 | -0.25 | 7.00 | 4.74 | 0.79 | 0.03 |
| **FCA229** | 4.00 | 3.82 | 0.65 | 0.56 | 6.00 | 4.26 | 0.72 | -0.03 | 6.00 | 3.96 | 0.74 | 0.19 | 4.00 | 3.97 | 0.71 | 0.38 | 7.00 | 4.01 | 0.63 | -0.03 |
| **FCA275** | 3.00 | 2.71 | 0.50 | -0.07 | 3.00 | 2.91 | 0.63 | -0.11 | 3.00 | 2.91 | 0.64 | 0.18 | 4.00 | 3.82 | 0.65 | 0.56 | 3.00 | 2.83 | 0.58 | 0.03 |
| **Mean** | 3.25 | 3.14 | 0.54 | 0.13 | 4.33 | 3.35 | 0.60 | -0.08 | 5.00 | 3.38 | 0.61 | 0.12 | 3.92 | 3.55 | 0.58 | -0.01 | 6.00 | 3.72 | 0.65 | 0.05 |
| ***SD*** | 0.97 | 0.97 | 0.16 | 0.30 | 1.72 | 1.02 | 0.16 | 0.14 | 2.26 | 1.13 | 0.22 | 0.15 | 1.31 | 1.05 | 0.11 | 0.28 | 2.17 | 0.89 | 0.11 | 0.11 |

**Table S1.** **Summary statistics of genetic diversity by locus for Mesoamerican jaguars.** Estimates of genetic diversity for jaguars (*n* = 115) detected across five Mesoamerican countries (Belize, Costa Rica, Guatemala, Honduras, Mexico), including number of alleles (*N_A_*), rarified allelic richness (*A_R_*), expected heterozygosity (*H_E_*), and inbreeding coefficient (*F_IS_*)_._ *n*, number of individual felids; *SD*, standard deviation.
