# Supplementary material for: Genetic Diversity and Population Structure of Mesoamerican Jaguars (Panthera onca): Implications for Conservation and Management
Source: PLoS One. 2016 Oct 26;11(10):e0162377. doi: 10.1371/journal.pone.0162377 (PMC5082669; doi:10.1371/journal.pone.0162377)
Supplement: S2 Table — P-value for the Hardy-Weinberg equilibrium (HWE) test (PHW), and frequency of null alleles (FNull) at 12 microsatellite loci for 115 jaguar samples collected across five Mesoamerican countries (Belize, Costa Rica, Guatemala, Honduras, Mexico). n, number of individuals. (DOCX) [file pone.0162377.s005.docx]

**Table S2**. **Hardy-Weinberg equilibrium and null allele analysis for Mesoamerican jaguars***. P*-value for the Hardy-Weinberg equilibrium (HWE) test (*P_HW_*), and frequency of null alleles (*F_Null_*) at 12 microsatellite loci for 115 jaguar samples collected across five Mesoamerican countries (Belize, Costa Rica, Guatemala, Honduras, Mexico). *n*, number of individuals.

| **Locus** | **Mexico** (*n* = 7) | | **Guatemala** (*n* = 15) | | **Belize** (*n* = 50) | | **Honduras** (*n* = 7) | | **Costa Rica** (*n* = 36) | |
| --- | --- | --- | --- | --- | --- | --- | --- | --- | --- | --- |
|  | ***P_HW_*** | ***F_Null_*** | ***P_HW_*** | ***F_Null_*** | ***P_HW_*** | ***F_Null_*** | ***P_HW_*** | ***F_Null_*** | ***P_HW_*** | ***F_Null_*** |
| **FCA032** | 0.72 | 0.10 | 0.93 | -0.12 | 0.28 | 0.07 | 1.00 | -0.38 | 0.73 | 0.05 |
| **FCA075** | 1.00 | 0.01 | 0.30 | 0.02 | 0.00 | 0.14 | 1.00 | -0.42 | 0.56 | -0.01 |
| **FCA096** | 0.25 | 0.01 | 0.91 | -0.03 | 0.42 | -0.04 | 0.51 | 0.05 | 0.02 | 0.09 |
| **FCA100** | 0.08 | 0.10 | 0.67 | -0.11 | 0.08 | 0.06 | 1.00 | -0.35 | 0.25 | 0.03 |
| **FCA124** | 0.78 | -0.05 | 0.36 | -0.17 | 0.40 | 0.07 | 0.55 | -0.07 | 0.12 | 0.11 |
| **FCA126** | 1.00 | -0.24 | 0.44 | -0.34 | 0.38 | -0.06 | 0.44 | -0.11 | 0.00 | 0.04 |
| **FCA132** | 1.00 | -0.37 | 1.00 | -0.08 | 1.00 | -0.04 | 0.44 | 0.07 | 0.09 | -0.04 |
| **FCA208** | 0.31 | 0.05 | 0.43 | 0.08 | 0.94 | -0.05 | 0.83 | -0.02 | 0.17 | 0.04 |
| **FCA212** | 0.27 | 0.11 | 0.58 | -0.40 | 0.01 | 0.15 | 1.00 | -0.09 | 0.49 | -0.11 |
| **FCA225** | 0.50 | -0.09 | 0.79 | 0.03 | 0.19 | 0.04 | 0.08 | -0.19 | 0.25 | 0.00 |
| **FCA229** | 0.10 | 0.10 | 0.25 | -0.05 | 0.02 | 0.09 | 0.19 | 0.16 | 0.68 | -0.01 |
| **FCA275** | 0.32 | -0.04 | 0.88 | -0.06 | 0.10 | 0.08 | 0.02 | 0.25 | 0.71 | 0.02 |
